# Reliability Analysis of Semiconductor Material Processing for Advanced Integrated Circuit Chip Manufacturing

**DOI:** 10.3390/mi17101131

**Published:** 2026-09-28

**Authors:** Yihan Zhang, Shan Jiang, Daqiang Zhang

**Affiliations:** 1 School of Electrical and Electronic Engineering, Nanyang Technological University, Singapore 639798, Singapore; yihan020@e.ntu.edu.sg; 2 School of Materials Science and Engineering, Nanyang Technological University, Singapore 639798, Singapore; asdfghjkljs112@gmail.com; 3 School of Software Engineering, Tongji University, Shanghai 201804, China

**Keywords:** semiconductor manufacturing, process reliability, uncertainty propagation, physics-informed modeling, yield optimization

## Abstract

Advanced integrated-circuit fabrication couples deposition, lithography, etching, implantation, annealing, and chemical–mechanical polishing, allowing local fluctuations to propagate into defects, electrical drift, yield loss, and premature failure. We develop a process-graph reliability framework combining physics-informed models, process-data analytics, and uncertainty quantification. A process–structure–property–reliability map is embedded in a directed fabrication graph, while a pathwise operator decomposes first-order uncertainty propagation into stage and cross-stage contributions. We derive sub-Gaussian and Wasserstein failure certificates, an independent-sample finite-data bound, and a risk-allocation scheme with explicit convergence conditions. In reproducible virtual-fab experiments, the cost-penalized implementation achieves 98.30% nominal yield and 82.2 kppm shifted failure. Under a matched-budget audit, all optimized policies fall within 81.2–82.5 kppm, indicating that the principal contribution is auditable cross-stage certification rather than unconditional empirical dominance.

## 1. Introduction

Advanced integrated-circuit fabrication comprises a sequence of strongly coupled material transformations rather than a set of independent unit operations. Thin-film deposition changes thickness, stress, composition, and interface roughness; lithography converts optical and thermal disturbances into critical-dimension and overlay errors; etching transfers those errors into three-dimensional geometry; and subsequent implantation, annealing, and chemical–mechanical polishing (CMP) alter dopant profiles, crystal damage, topography, and defect populations. Recent studies have highlighted the increasing importance of controllable etch physics, thermally stable projection lithography, and atomic-scale process integration as feature dimensions approach a few nanometers [1,2,3]. The reliability consequence is that a locally acceptable deviation may become harmful after it interacts with downstream material states.

Advanced processing introduces uncertainty across multiple scales. Plasma chemistry acts on microsecond time scales, wafer temperature evolves over seconds or minutes, consumables age over hundreds of lots, and latent defects may manifest after billions of electrical switching cycles. Atomic-layer etching studies show that small changes in adsorption, ion energy, and surface reaction pathways can change selectivity and damage [4]. At the same time, photoresist properties and microfabrication routes determine pattern fidelity before etch transfer, while micro- and nanochannel fabrication research illustrates how geometry, surface condition, and process sequence interact across scales [5,6]. A reliability model must therefore preserve causal process order and retain nonlinear interaction terms instead of averaging them away.

Metrology and process-control data provide a complementary view of these mechanisms. Multiplexed optical targets can connect pattern-shift measurements to electrical properties, and artificial intelligence-assisted optical metrology can accelerate inverse reconstruction for high-aspect-ratio structures [7,8]. Nevertheless, data from a production line are heterogeneous, censored, and often sparse near the failure boundary. Recipe changes, maintenance actions, tool matching, sampling policies, and product mix create distribution shifts. A predictor that interpolates well on historical wafers may be poorly calibrated when the process window changes. These conditions favor combining physical structure with data-driven uncertainty quantification rather than relying on a purely black-box reliability score.

Machine learning is increasingly used for semiconductor process optimization, yield prediction, virtual metrology, and root-cause diagnosis. A recent systematic review organized this literature by process task and learning paradigm [9]. Fast ramp-up strategies use production data to identify influential factors and shorten yield-learning cycles, whereas hierarchical virtual-metrology methods explicitly address multimodal data from multiple batches and process stages [10,11]. Although these methods improve predictive performance, most are evaluated primarily in terms of predictive accuracy or expected loss. They do not provide a stage-resolved certificate that upper-bounds final failure probability when correlated disturbances propagate across the fabrication chain.

CMP illustrates this gap particularly clearly. Its material removal depends on pad state, pressure zones, relative velocity, slurry chemistry, temperature, and incoming topography. Recent reviews and studies using production-fab data show that machine learning can predict removal rate and within-wafer non-uniformity, while also highlighting limited transferability, explainability, and robustness under product changes [12,13]. Because CMP is a late-stage process, an apparent CMP anomaly may originate from upstream deposition or lithography. A reliable diagnostic method must distinguish direct CMP variability from inherited variability and must quantify how much each causal path contributes to final electrical and lifetime risk.

Digital twin and yield classification studies have begun to combine process optimization with virtual representations of semiconductor equipment. Bayesian optimization has been integrated with deep learning for process control, and regional wafer-yield models have been designed for imbalanced low-yield data [14,15]. Regression and predictive-modeling reviews further show that wafer manufacturing increasingly relies on hybrid statistical and machine learning pipelines [16]. However, a digital twin without an explicit risk envelope can recommend a set point that is optimal for the modeled distribution yet unsafe under a plausible process shift. Reliability-oriented optimization therefore requires a certificate whose assumptions, confidence level, and degree of conservatism are explicit.

Recent research also addresses high-dimensional overlay control, wafer-defect classification, and sparse-data defect discovery. Tensor-based process control handles unstable image disturbances and provides controller stability conditions [17]. Comprehensive defect-classification evaluations and few-shot subclass discovery improve the interpretation of wafer-map patterns when labeled failures are scarce [18,19]. A hierarchical attention network has also been used to connect production resources to yield outcomes [20]. These advances motivate graph-structured representations, but they generally treat graph structure as a predictive architecture. In contrast, the fabrication graph in this paper has a physical meaning: its edges transport material-state perturbations, uncertainty, and risk contributions.

Industrial deployment must also cope with rare failures, equipment faults, evolving production priorities, and limited labels. Two-step learning has recently been used for industrial yield prediction with complex production histories [21]. Temporal raw-trace augmentation improves fault detection under defective-wafer imbalance, while ontology-driven Bayesian networks connect fault causes to corrective actions [22,23]. Data-driven lot-priority models quantify cycle-time effects, and proactive equipment-monitoring optimization links real-time tool signals to end-of-line yield [24,25]. A recent artificial intelligence survey of wafer-yield prediction further clarifies the remaining data and modeling challenges [26]. These approaches are valuable, but they do not establish a mathematical link from process fluctuations to microstructural defects, electrical drift, and lifetime failure.

The need for a cross-stage formulation is reinforced by 2025–2026 studies of lot cycle time and temporally accumulated production histories. Deep clustering has improved cycle-time prediction under changing fab conditions, while temporal yield models show that cumulative exposure can reveal degradation missed by equipment identifiers alone [27,28]. Motivated by these observations, this paper develops a process–structure–property–reliability (PSPR) operator. The novelty is not the use of a graph representation by itself. It is the integration of (i) an ordered pathwise Jacobian operator that assigns downstream reliability sensitivity to physical fabrication paths, (ii) sub-Gaussian and Wasserstein failure certificates stated on the same reliability margin, and (iii) a certificate-compatible risk-allocation layer whose empirical claims are audited at equal intervention effort. We therefore avoid claiming that graph-based process reasoning is itself new.

Table 1 positions these components against representative recent semiconductor and manufacturing methods already discussed above. The comparison distinguishes prediction or optimization functions from an explicit pathwise reliability certificate; it is not intended as a performance ranking because the cited studies use different data, processes, and endpoints.

For readability, the abbreviations used repeatedly below are defined here: process graph (PG), reliability certificate allocation (RCA), sequential quadratic programming (SQP), Monte Carlo (MC), Dvoretzky–Kiefer–Wolfowitz (DKW), and cumulative distribution function (CDF).

**Figure 1:**
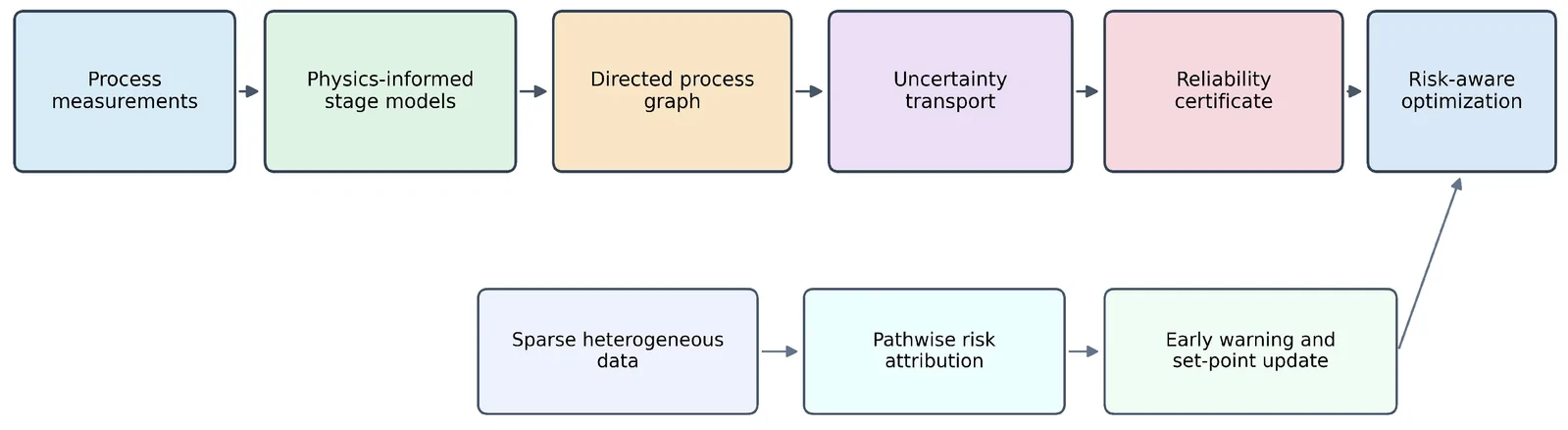
Process-graph reliability analysis workflow.

The remainder of the paper follows the qualification logic in Figure 1. Section 2 first defines the fabrication graph and PSPR margin, then derives pathwise uncertainty transport and reliability certificates, and finally introduces the certificate-oriented optimization and the reproducible virtual-fab design. Section 3 begins with pathwise and lifetime diagnostics; separates cost-penalized performance from the matched-budget fairness audit; and then presents stress, finite-sample, topology, parameter, and flow-length audits, before discussing engineering limitations and deployment gates. Section 4 summarizes the evidence boundary and the industrial validation steps still required.

Table 2 lists the principal symbols used throughout this paper. The notation is intentionally compact so that process, uncertainty, and reliability quantities remain easy to trace across sections.

## 2. Materials and Methods

### 2.1. Process–Structure–Property–Reliability Formulation

#### 2.1.1. Directed Fabrication Graph

Let G=(V,E) be a directed acyclic graph with ordered vertices V={1,…,K}. Each vertex denotes a material-processing stage, and an edge (j,k)∈E means that the post-stage state at *j* is a physical input to stage *k*. For the representative chain considered here, K=6 and the vertices correspond to deposition, lithography, etching, ion implantation, annealing, and CMP. A state $x_{k}\in R^{d_{k}}$ contains normalized descriptors such as thickness deviation, residual stress, critical-dimension error, sidewall angle, damage density, dopant-profile error, roughness, and non-uniformity. The causal recursion is

(1) $$x_{k}=f_{k}\;\left(x_{\mathrm{pa}(k)},u_{k};\theta_{k}\right)+G_{k}\varepsilon_{k},\;k=1,\ldots ,K,$$

where $x_{\mathrm{pa}(k)}$ concatenates parent states, $u_{k}\in U_{k}$ is a bounded intervention variable (for example, a normalized recipe correction or mitigation intensity), $\theta_{k}$ is a physical parameter vector, and $G_{k}\varepsilon_{k}$ represents unresolved fluctuation. The process trajectory is $x={\left(x_{1}^{⊤},\ldots ,x_{K}^{⊤}\right)}^{⊤}$.

Figure 1 summarizes the analysis workflow; it is a feed-forward qualification sequence rather than a feedback-control loop. Measurements and physical models define the process graph, uncertainty is transported through directed paths, the resulting risk is certified, and a risk-aware optimizer updates allowable process windows. The lower branch separates sparse heterogeneous data from pathwise attribution, ensuring that data scarcity does not erase known physical couplings.

The stage maps in (1) need not share the same functional form. A deposition model can combine nucleation kinetics, precursor transport, and wafer-temperature dependence. A lithography model can combine dose, focus, resist chemistry, and thermal deformation. An etch model can use ion-flux and surface-reaction terms. An implantation–annealing pair can represent dose straggle, point-defect evolution, activation, and diffusion. A CMP model can combine pressure, relative velocity, slurry state, and incoming topography. The framework allows different state dimensions and model classes; it requires only differentiability within a neighborhood of the qualified process window.

#### 2.1.2. PSPR Map and Reliability Margin

The final structural descriptor is generated by

(2) $$z=h_{s}\left(x_{1},\ldots ,x_{K}\right),$$

where $h_{s}$ aggregates geometric, compositional, interfacial, and defect-related states. We define a nonnegative defect functional D(z) and a nonnegative electrical-drift magnitude P(z) as

(3) $$\begin{array}{cc} D(z) & =d_{0}+\sum_{k=1}^{K}a_{k}\phi_{k}\left(x_{k}\right)+\sum_{(i,j)\in E}a_{ij}\phi_{ij}\left(x_{i},x_{j}\right), \end{array}$$

(4) $$\begin{array}{cc} P(z) & =p_{0}+\sum_{k=1}^{K}b_{k}\psi_{k}\left(x_{k}\right)+\sum_{p\in P_{\;\mathrm{crit}}}b_{p}\psi_{p}\left(x_{p}\right), \end{array}$$

where $P_{\;\mathrm{crit}}$ is a set of physically important directed paths. The interaction terms include, for example, lithography–etch pattern transfer, implantation–annealing activation, and etch–CMP topography coupling. The signed reliability margin is

(5) $$M\left(x,u\right)=m_{\mathrm{spec}}-c_{D}D\left(z\right)-c_{P}P\left(z\right)-c_{L}L\left(z\right),$$

where $m_{\mathrm{spec}}>0$ is a specification allowance, $c_{D},c_{P},c_{L}\geq 0$, and L(z) is an optional lifetime-degradation term when an independently calibrated lifetime model is available. A wafer is classified as reliable when M>0 and failed when M≤0. In the virtual-fab experiment below, $c_{L}$ is set to zero and the synthetic equivalent-cycle index is generated downstream from *M*; this avoids using a derived lifetime outcome circularly inside the margin that generates it.

**Figure 2:**
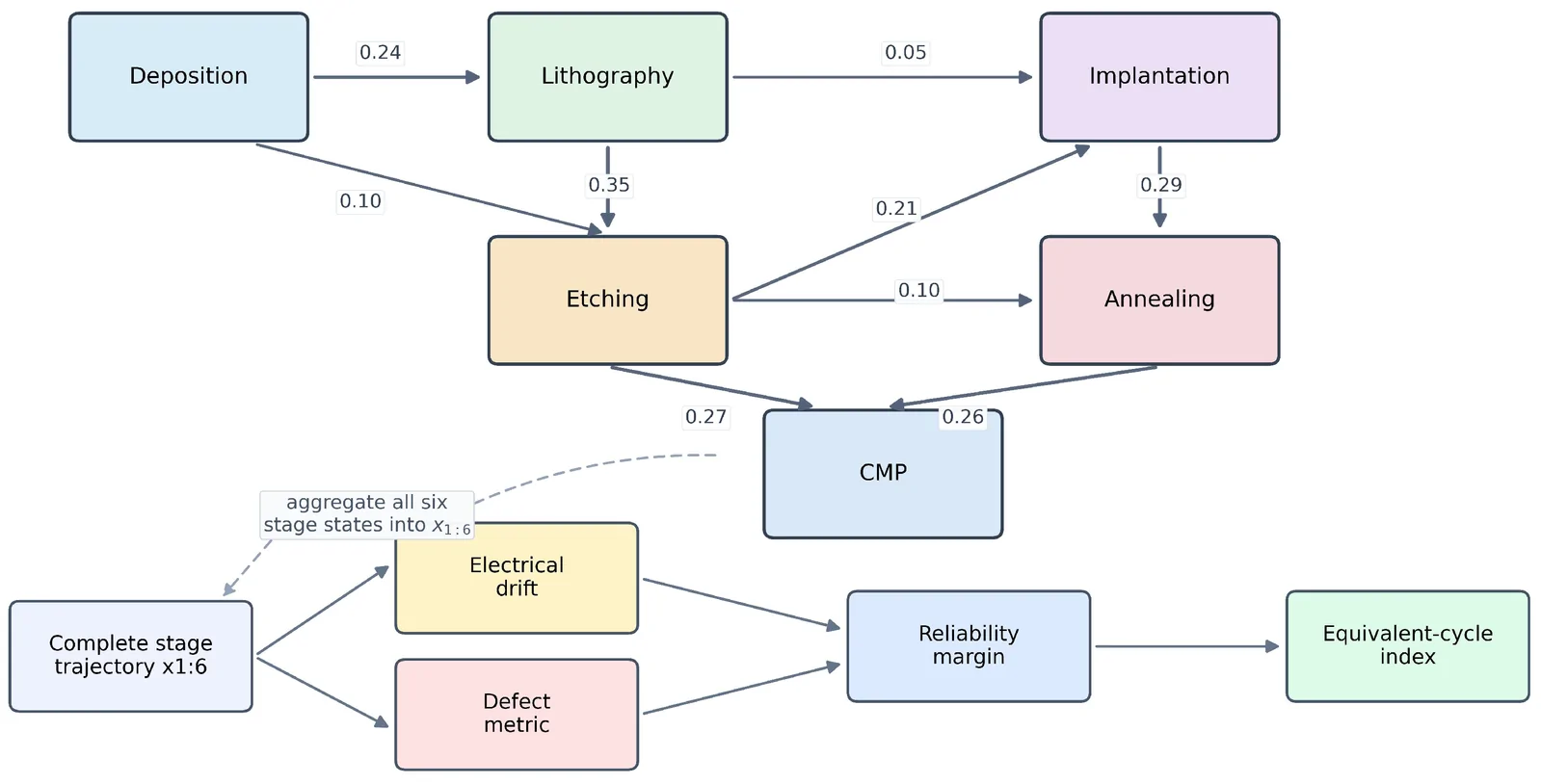
Directed fabrication graph and PSPR pathway. Solid arrows indicate directed process/material or diagnostic propagation; the dashed aggregation link associates the upper fabrication graph with the complete stage trajectory $x_{1:6}$, which then forms the defect and electrical diagnostics.

Figure 2 shows the representative directed graph. The upper panel shows process stages and selected direct couplings; the displayed edge values are local one-step coefficients rather than entries of the total path-sum transport operator. Products and sums of these coefficients generate downstream transport along all directed paths. The lower pathway shows how the complete stage trajectory $x_{1:6}$ enters the synthetic PSPR map. A dashed aggregation link connects the fabrication graph to $x_{1:6}$; the trajectory then feeds the defect and electrical terms, which jointly determine the reliability margin, and the equivalent-cycle index is generated downstream from that margin. Arrow endpoints terminate at the box boundaries to keep the transformations visually unambiguous.

**Assumption** **1** (Qualified process window)**.**
*For every k, the set $U_{k}$ is nonempty, compact, and convex. There is a closed reachable state set $X_{k}$ such that the admissible recursion remains in $X_{k}$ almost surely. The map $f_{k}$ is continuously differentiable on the product of its parent reachable sets and $U_{k}$, with uniformly bounded state and control Jacobians. The disturbances $\varepsilon_{k}$ possess finite second moments; any predictable conditional mean is absorbed into the corresponding stage map $f_{k}$. The functionals D, P, and L are continuous and grow at most quadratically on the reachable state sets. Whenever a pathwise gradient is invoked, these functionals are assumed differentiable at the nominal trajectory under consideration; locally Lipschitz nonsmooth terms are handled only at differentiability points or by a smooth local approximation.*

**Assumption** **2** (Stagewise incremental stability)**.**
*For each edge (j,k)∈E, there exists $L_{kj}\geq 0$ such that*

(6) $$∥f_{k}\left(v,u_{k};\theta_{k}\right)-f_{k}\left(w,u_{k};\theta_{k}\right)∥\leq \sum_{j\in \mathrm{pa}(k)}L_{kj}∥v_{j}-w_{j}∥.$$

*The weighted path gain satisfies*

(7) $$\Gamma =\max_{k}\sum_{p:\;p\to k}\prod_{(i,j)\in p}L_{ji}<\infty .$$

**Theorem** **1** (Well-posedness and trajectory stability)**.**
*Under Assumptions 1 and 2, Equation (1) defines a unique measurable process trajectory for every admissible control u and disturbance realization. For two trajectories generated by (u,ε) and $\left(\tilde{u},\tilde{\varepsilon }\right)$, there exist finite constants $C_{u}$ and $C_{\varepsilon }$ determined by directed path gains such that*

(8) $$\max_{1\leq k\leq K}∥x_{k}-{\tilde{x}}_{k}∥\leq C_{u}\max_{k}∥u_{k}-{\tilde{u}}_{k}∥+C_{\varepsilon }\max_{k}∥\varepsilon_{k}-{\tilde{\varepsilon }}_{k}∥.$$

*Consequently, the PSPR margin M(x,u) is continuous and integrable on the qualified process window.*

**Proof.**  Because G is acyclic, its vertices admit a topological ordering. At the first vertex, Equation (1) directly defines a unique measurable $x_{1}$ from $\left(u_{1},\varepsilon_{1}\right)$. Suppose that all states preceding vertex *k* are uniquely defined and measurable. Then, the parent vector $x_{\mathrm{pa}(k)}$ is measurable, and continuity of $f_{k}$ implies that $x_{k}$ is also uniquely defined and measurable. Induction over the topological order proves existence and uniqueness for the complete trajectory.

For stability, define $\delta_{k}=∥x_{k}-{\tilde{x}}_{k}∥$. The uniformly bounded Jacobians in Assumption 1 give a finite control Lipschitz constant $B_{k}$ and disturbance gain $\eta_{k}=∥G_{k}∥$, so

(9) $$\delta_{k}\leq \sum_{j\in \mathrm{pa}(k)}L_{kj}\delta_{j}+B_{k}∥u_{k}-{\tilde{u}}_{k}∥+\eta_{k}∥\varepsilon_{k}-{\tilde{\varepsilon }}_{k}∥.$$

Recursively substitute the corresponding inequalities for every parent. Each substitution creates one directed path terminating at *k*. Since the graph is finite and acyclic, only finitely many paths occur. Collecting the products of edge gains yields

(10) $$\delta_{k}\leq \sum_{r⪯k}\left(\sum_{p:r\to k}\prod_{(i,j)\in p}L_{ji}\right)\left[B_{r}∥u_{r}-{\tilde{u}}_{r}∥+\eta_{r}∥\varepsilon_{r}-{\tilde{\varepsilon }}_{r}∥\right].$$

Taking the maximum over *k* and using finiteness of the path sums produces (8). The same recursion, together with finite second moments of the disturbances and bounded Jacobians on the reachable sets, implies by induction that every $x_{k}$ has a finite second moment. Since *D*, *P*, and *L* have at most quadratic growth, the margin is integrable. Continuity follows from composition of the stage and PSPR maps.    □

**Remark** **1.** *Beyond establishing existence, the theorem identifies the mathematical object that governs cross-stage amplification: products of local gains along directed fabrication paths. A stage with modest local sensitivity can dominate final reliability when it lies upstream of several high-gain transformations. This observation motivates the pathwise uncertainty transport developed next and explains why independent-stage tolerancing can systematically underestimate downstream risk.*

### 2.2. Pathwise Uncertainty Transport and Reliability Certification

#### 2.2.1. Directed Sensitivity Operator

Let $J_{k\leftarrow j}=\partial x_{k}/\partial x_{j}$ denote a local Jacobian for an edge (j,k). For a directed path $p=(v_{0},v_{1},\ldots ,v_{q})$, define the ordered path operator

(11) $$\Pi_{p}=J_{v_{q}\leftarrow v_{q-1}}J_{v_{q-1}\leftarrow v_{q-2}}\cdots J_{v_{1}\leftarrow v_{0}}.$$

If the disturbance entering stage *j* is $\xi_{j}=G_{j}\varepsilon_{j}$, then the first-order contribution of $\xi_{j}$ to a downstream state $x_{k}$ is

(12) $$\Delta x_{k|j}=\sum_{p:j\to k}\Pi_{p}\xi_{j}.$$

The sum covers all directed paths from *j* to *k*, including the identity path when j=k. This operator preserves both direct and indirect effects. It also distinguishes two stages that have similar marginal variance but different downstream connectivity.

For the final margin, let $g_{k}\left(x,u\right)=\nabla_{x_{k}}M\left(x,u\right)$; both state and intervention dependence are kept explicit. The stage-to-margin sensitivity is

(13) $$s_{j}=\sum_{k⪰j}{\left(\sum_{p:j\to k}\Pi_{p}\right)}^{\;⊤}g_{k}.$$

A large $∥s_{j}∥$ indicates that small perturbations at stage *j* strongly affect reliability. The direction of $s_{j}$ indicates how a local correction changes the margin.

**Proposition** **1** (Pathwise covariance decomposition)**.**
*Let $\Sigma_{j\ell }=\mathrm{Cov}\left(\xi_{j},\xi_{\ell }\right)$ denote the blocks of the joint covariance matrix of the stage disturbances. Under the first-order linearization of Equation (1), with the Jacobians and margin gradients evaluated and frozen along a nominal trajectory, the covariance of the first-order downstream perturbation $\Delta x_{k}$ is*

(14) $$\Sigma_{x_{k}}=\sum_{j⪯k}\sum_{\ell ⪯k}T_{kj}\Sigma_{j\ell }T_{k\ell }^{⊤},\;T_{kj}=\sum_{p:j\to k}\Pi_{p}.$$

*The corresponding first-order variance of the reliability margin is*

(15) $$\sigma_{M}^{2}=\sum_{j=1}^{K}\sum_{\ell =1}^{K}s_{j}^{⊤}\Sigma_{j\ell }s_{\ell }.$$

*If the stage disturbances are mutually uncorrelated, these expressions reduce to the diagonal sums $\Sigma_{x_{k}}=\sum_{j⪯k}T_{kj}\Sigma_{jj}T_{kj}^{⊤}$ and $\sigma_{M}^{2}=\sum_{j}s_{j}^{⊤}\Sigma_{jj}s_{j}$.*

**Proof.**  Stack the disturbance vectors as $\xi ={\left(\xi_{1}^{⊤},\ldots ,\xi_{K}^{⊤}\right)}^{⊤}$, and let $\Sigma_{\xi }={\left[\Sigma_{j\ell }\right]}_{j,\ell =1}^{K}$. Linearizing the acyclic recursion and collecting every path from source *j* to target *k* give

(16) $$\Delta x_{k}=\sum_{j⪯k}T_{kj}\xi_{j}=T_{k}\xi ,\;T_{k}=\left[T_{k1}\;\cdots \;T_{kK}\right],$$

where blocks with j⋠k are zero. Therefore,

(17) $$\mathrm{Cov}\left(\Delta x_{k}\right)=T_{k}\Sigma_{\xi }T_{k}^{⊤}=\sum_{j⪯k}\sum_{\ell ⪯k}T_{kj}\Sigma_{j\ell }T_{k\ell }^{⊤},$$

which proves Equation (14) without requiring independence. The linearized margin is $\Delta M=\sum_{j}s_{j}^{⊤}\xi_{j}=s^{⊤}\xi$, with stacked sensitivity $s={\left(s_{1}^{⊤},\ldots ,s_{K}^{⊤}\right)}^{⊤}$. Hence, $\mathrm{Var}\left(\Delta M\right)=s^{⊤}\Sigma_{\xi }s$, and expanding the block product gives Equation (15). When $\Sigma_{j\ell }=0$ for j≠ℓ, all cross terms vanish and the stated diagonal formulas follow.    □

#### 2.2.2. Sub-Gaussian Cross-Stage Failure Bound

Covariance alone does not characterize rare-event risk. We therefore derive a non-asymptotic tail certificate. Let $F_{k}$ be the filtration generated by all disturbances through stage *k*. Define the conditional margin increment

(18) $$\Delta_{k}=E\left[M|F_{k}\right]-E\left[M|F_{k-1}\right],\;M-E\left[M\right]=\sum_{k=1}^{K}\Delta_{k}.$$

**Assumption** **3** (Conditional sub-Gaussian transport)**.**
*For every k and all t∈R,*

(19) $$E\left[\exp\left(t\Delta_{k}\right)|F_{k-1}\right]\leq \exp\;\left(\frac{t^{2}\nu_{k}^{2}\left(u\right)}{2}\right),$$

*where $\nu_{k}\left(u\right)$ is an upper bound obtained from stage-noise scale and directed path gain.*

**Theorem** **2** (Cross-stage reliability certificate)**.**
*Let $\mu_{M}\left(u\right)=E\left[M\left(x,u\right)\right]$. Under Assumption 3, the final failure probability satisfies*

(20) $$P\left\{M\leq 0\right\}\leq \exp\;\left[-\frac{{\left(\mu_{M}\left(u\right)\right)}_{+}^{2}}{2\sum_{k=1}^{K}\nu_{k}^{2}\left(u\right)}\right]=:C_{\mathrm{SG}}\left(u\right),$$

*where ${\left(a\right)}_{+}=\max\left\{a,0\right\}$ and the displayed expression applies when $V\left(u\right)=\sum_{k}\nu_{k}^{2}\left(u\right)>0$. If V(u)=0, define $C_{\mathrm{SG}}\left(u\right)=0$ for $\mu_{M}\left(u\right)>0$ and $C_{\mathrm{SG}}\left(u\right)=1$ otherwise. If $\mu_{M}\left(u\right)>0$ and V(u)>0, increasing the mean margin or decreasing any positive transported stage scale $\nu_{k}$ strictly tightens the bound.*

**Proof.**  Failure implies $M-\mu_{M}\leq -\mu_{M}$. For any t>0, Markov’s inequality gives

(21) $$\begin{array}{cc} P(M-\mu_{M}\leq -\mu_{M}) & =P\;\left(\exp\left[-t\left(M-\mu_{M}\right)\right]\geq \exp\left(t\mu_{M}\right)\right)\\ \; & \leq \exp\left(-t\mu_{M}\right)E\exp\left[-t\left(M-\mu_{M}\right)\right]. \end{array}$$

Using the martingale representation (18) and the tower property,

(22) $$\begin{array}{cc} E\exp\;\left(-t\sum_{k=1}^{K}\Delta_{k}\right) & =E\left[\exp\;\left(-t\sum_{k=1}^{K-1}\Delta_{k}\right)E\;\left(e^{-t\Delta_{K}}|F_{K-1}\right)\right]\\ \; & \leq \exp\;\left(\frac{t^{2}\nu_{K}^{2}}{2}\right)E\exp\;\left(-t\sum_{k=1}^{K-1}\Delta_{k}\right). \end{array}$$

Repeating this argument backward through stage 1 yields

(23) $$E\exp\left[-t\left(M-\mu_{M}\right)\right]\leq \exp\;\left(\frac{t^{2}}{2}\sum_{k=1}^{K}\nu_{k}^{2}\right).$$

Substituting (23) into (21) produces

(24) $$P\left(M\leq 0\right)\leq \exp\;\left[-t\mu_{M}+\frac{t^{2}}{2}\sum_{k=1}^{K}\nu_{k}^{2}\right].$$

When $V=\sum_{k}\nu_{k}^{2}>0$ and $\mu_{M}>0$, the right-hand side is minimized by $t^{★}=\mu_{M}/V$. Substitution gives (20). When V>0 and $\mu_{M}\leq 0$, the probability bound 1 is recovered through ${\left(\mu_{M}\right)}_{+}=0$. If V=0, the conditional moment bound forces every martingale increment $\Delta_{k}$ to vanish almost surely, so $M=\mu_{M}$ almost surely and the stated convention is exact. Strict monotonicity in the nondegenerate case follows by direct differentiation of the exponent.    □

#### 2.2.3. Finite-Data Distributionally Robust Certificate

The sub-Gaussian certificate relies on transported scale estimates. To guard against sampling uncertainty and moderate distribution shift, let ${\hat{P}}_{n}=n^{-1}\sum_{i=1}^{n}\delta_{\zeta_{i}}$ be the empirical distribution of wafer records $\zeta_{i}$ on a common closed qualification domain Z. Define the support-restricted Wasserstein ambiguity set

(25) $$B_{n}\left(\rho \right)=\left\{Q:\mathrm{supp}\left(Q\right)\subseteq Z,\;W_{1}\left(Q,{\hat{P}}_{n}\right)\leq \rho \right\}.$$

For δ>0, introduce the smooth bounded majorant

(26) $$\ell_{\delta }\left(m\right)=\frac{2}{1+\exp(m/\delta )}.$$

It satisfies $1_{\left\{m\leq 0\right\}}\leq \ell_{\delta }\left(m\right)\leq 2$ and is globally 1/(2δ)-Lipschitz. Unlike an exponential surrogate, it requires no artificial global lower bound on the reliability margin.

**Theorem** **3** (Wasserstein robust failure certificate)**.**
*Assume that M(ζ,u) is $L_{M}\left(u\right)$-Lipschitz in ζ on Z. Then, for every $Q\in B_{n}\left(\rho \right)$ and every δ>0,*

(27) $$Q\left(M\leq 0\right)\leq \min\;\left\{1,\frac{1}{n}\sum_{i=1}^{n}\ell_{\delta }\left(M\left(\zeta_{i},u\right)\right)+\frac{\rho L_{M}\left(u\right)}{2\delta }\right\}.$$

*Consequently, taking the infimum over any fixed compact interval $\left[\delta_{\min},\delta_{\max}\right]$ contained in (0,∞) preserves a valid robust upper bound. If a concentration result supplies $\rho_{n}\left(\beta \right)$ such that the unknown production distribution belongs to $B_{n}\left(\rho_{n}\left(\beta \right)\right)$ with probability at least 1−β, this bound is a 1−β finite-data certificate [29].*

**Proof.**  The pointwise majorization in Equation (26) gives

(28) $$Q\left(M\leq 0\right)\leq E_{Q}\left[\ell_{\delta }\left(M\right)\right].$$

Differentiating $\ell_{\delta }$ yields

(29) $$\left|\ell_{\delta }^{\prime }\left(m\right)\right|=\frac{2e^{m/\delta }}{\delta {\left(1+e^{m/\delta }\right)}^{2}}\leq \frac{1}{2\delta }.$$

Hence, $\ell_{\delta }\circ M$ is $L_{M}\left(u\right)/\left(2\delta \right)$-Lipschitz. For any coupling π of *Q* and ${\hat{P}}_{n}$,

(30) $$\begin{array}{cc} E_{Q}\left[\ell_{\delta }\left(M\right)\right]-E_{{\hat{P}}_{n}}\left[\ell_{\delta }\left(M\right)\right]\; & \leq \frac{L_{M}\left(u\right)}{2\delta }\int ∥\zeta -\tilde{\zeta }∥\;d\pi . \end{array}$$

Taking the infimum over couplings gives $W_{1}\left(Q,{\hat{P}}_{n}\right)$, which is at most ρ. The empirical expectation is the first term in Equation (27); substitution into Equation (28), together with the trivial probability bound Q(M≤0)≤1, proves the clipped bound. Since every fixed δ gives a valid upper bound, their infimum over a fixed interval is also valid. The confidence statement follows on the event that the unknown distribution lies in the ambiguity set.    □

**Corollary** **1** (Independent-sample finite-data bound)**.**
*Let a policy u be selected without using a certification sample of $n_{c}$-independent and identically distributed margins $M_{1},\ldots ,M_{n_{c}}$ with cumulative distribution function $F_{u}$. The certification sample is independent of all data used to select u. Define ${\hat{F}}_{n_{c},u}\left(m\right)=n_{c}^{-1}\sum_{i=1}^{n_{c}}1_{\left\{M_{i}\leq m\right\}}$. Then, with probability at least 1−β,*

(31) $$P\left(M\leq 0\right)\leq {\hat{F}}_{n_{c},u}\left(0\right)+\epsilon_{n_{c}}\left(\beta \right),\;\epsilon_{n_{c}}\left(\beta \right)=\sqrt{\frac{\log(2/\beta )}{2n_{c}}}.$$

*If the scalar-margin distribution is supported on $\left[M_{-},M_{+}\right]$, then the first-order Wasserstein distance between the true and empirical scalar-margin distributions is at most $\left(M_{+}-M_{-}\right)\epsilon_{n_{c}}\left(\beta \right)$ on the same event.*

**Proof.**  Massart’s sharp-constant form of the Dvoretzky–Kiefer–Wolfowitz inequality [30] states that

(32) $$P\;\left(\underset{m}{\sup}\left|{\hat{F}}_{n_{c},u}\left(m\right)-F_{u}\left(m\right)\right|>\epsilon \right)\leq 2e^{-2n_{c}\epsilon^{2}}.$$

Setting the right-hand side to β and evaluating the resulting uniform event at m=0 proves Equation (31). In one dimension, $W_{1}$ equals the integral of the absolute difference between the two cumulative distribution functions. On $\left[M_{-},M_{+}\right]$, that integral is bounded by the interval length times the uniform CDF error, proving the final statement.    □

The DKW result is retained because it controls the entire scalar-margin CDF uniformly and therefore remains valid for threshold queries chosen after certification. For a single pre-specified failure threshold, binomial intervals can be substantially tighter. Section 3.4.1 therefore reports Wilson, exact Clopper–Pearson, percentile-bootstrap, and DKW upper limits side by side and treats DKW as a conservative qualification envelope rather than the preferred estimator of a single failure proportion.

**Figure 3:**
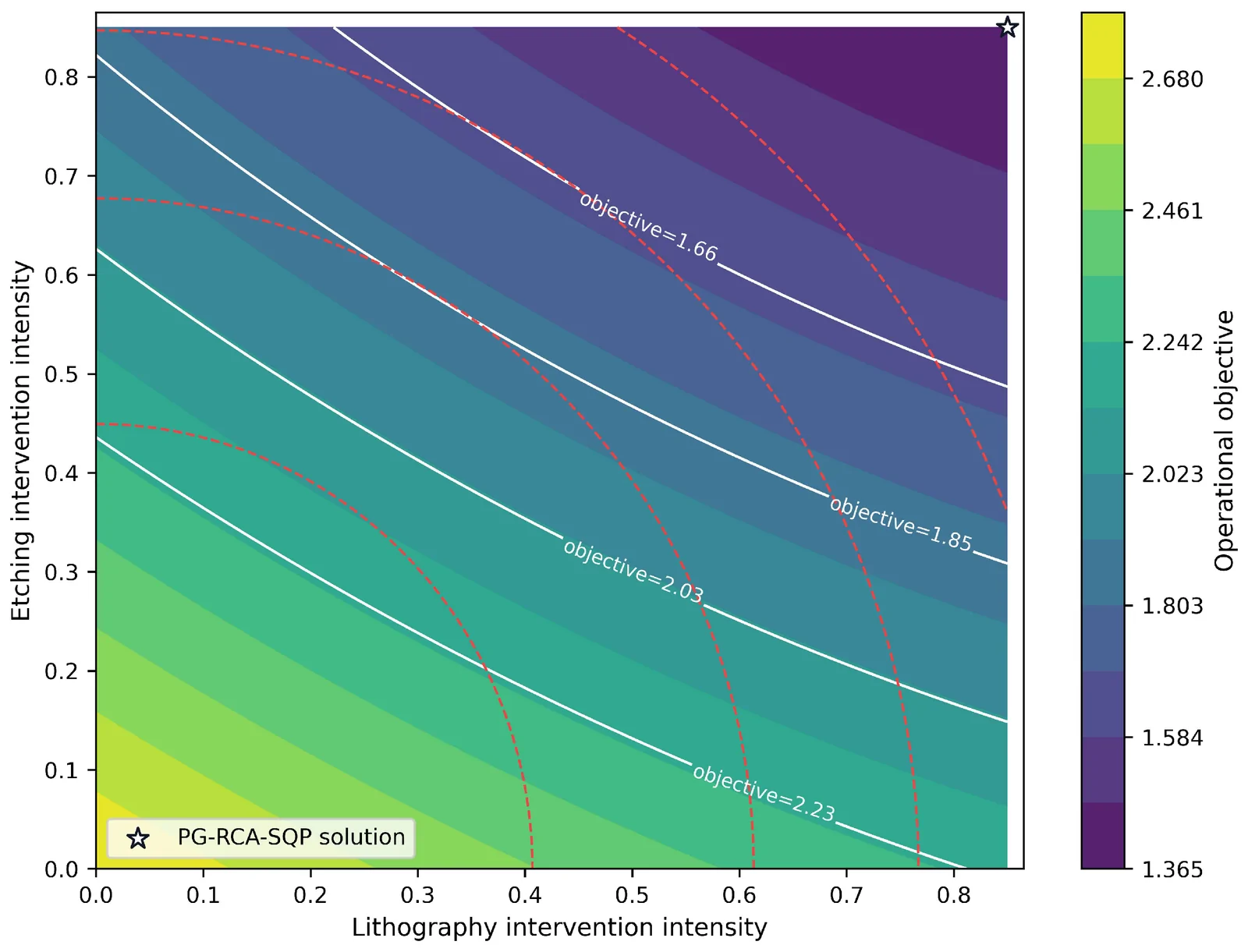
Feasible operational objective slice for lithography and etching intervention intensities.

Figure 3 illustrates a two-dimensional slice of the operational objective in Equation (36) over the qualified lithography and etching windows, with the other four controls fixed at their optimized values. The colored region contains the scenario-tail, transported-sensitivity, path-prior, and intervention-cost terms with the same weights used during optimization. Gray points exceed the common quadratic intervention budget and are excluded. White contours denote equal values of the feasible objective, dashed contours show intervention cost, and the star marks the PG-RCA-SQP solution. The plotted objective remains an operational score rather than a probability certificate.

**Remark** **2.** *The two certificates address complementary engineering needs. The sub-Gaussian bound exposes how stagewise disturbance scales accumulate through the graph, which is useful for tolerance allocation and mechanism analysis. The Wasserstein bound protects against limited sampling and moderate distribution shift, which is useful during ramp-up or product transfer. Their common input is the same PSPR margin, so physical interpretation is retained while statistical conservatism is adjusted to data availability.*

### 2.3. Reliability-Oriented Process Optimization

#### 2.3.1. Risk Allocation Problem

Let $c_{k}\left(u_{k}\right)$ denote the cost of applying the intervention at stage *k*. It may represent throughput loss, consumable use, energy, tool qualification effort, or distance from a productivity-optimal set point. We define the robust reliability objective

(33) $$R\left(u\right)=\underset{\delta \in [\delta_{\min},\delta_{\max}]}{\inf}\left\{\frac{1}{n}\sum_{i=1}^{n}\ell_{\delta }\left(M\left(\zeta_{i},u\right)\right)+\frac{\rho_{n}\left(\beta \right)L_{M}\left(u\right)}{2\delta }\right\}.$$

For finite-dimensional computation, let s=1,…,S index prespecified training scenarios, where s=1 is the empirical sample and the remaining scenarios are physically admissible perturbations constructed without held-out data. At a fixed temperature τ>0, define

(34) $$r_{s}\left(u\right)=\tau \log\left[\frac{1}{n}\sum_{i=1}^{n}\exp\left\{-M\left(\zeta_{i}^{\left(s\right)},u\right)/\tau \right\}\right],$$

(35) $${\tilde{R}}_{\kappa }\left(u\right)=\frac{1}{\kappa }\log\left[\sum_{s=1}^{S}\exp\left\{\kappa r_{s}\left(u\right)\right\}\right],\;\kappa >0.$$

The operational score used in the virtual-fab study is

(36) $$\tilde{J}\left(u\right)=a_{R}{\tilde{R}}_{\kappa }\left(u\right)+a_{L}\rho {\hat{L}}_{M}\left(u\right)+a_{G}p_{G}\left(u\right)+\lambda_{c}c\left(u\right),$$

where ${\hat{L}}_{M}$ is a conservative transported local-sensitivity proxy and $p_{G}$ penalizes leaving high-gain paths weakly protected. Specifically, $g_{k}\left(x_{i},u\right)=\nabla_{x_{k}}M\left(x_{i},u\right)$ is the wafer-level margin gradient defined above, while ${\bar{g}}_{k}\left(u\right)=n^{-1}\sum_{i}\hat{|g_{k}\left(x_{i},u\right)|}$ is the componentwise sample mean of the triangle-inequality upper proxy used by the executable. In the executable study, if $\bar{g}\left(u\right)\geq 0$ denotes this vector, then

(37) $${\hat{L}}_{M}\left(u\right)={∥{\left|T\right|}^{⊤}\bar{g}\left(u\right)∥}_{2}.$$

For the synthetic state map x=Tdiag(1−0.55u)ξ, the direct innovation-to-margin gradient also contains diag(1−0.55u). Because every attenuation factor lies in [0,1], omitting that diagonal factor in Equation (37) deliberately gives a componentwise conservative upper proxy rather than an exact gradient norm; the control dependence that remains enters through $\bar{g}\left(u\right)$. In the virtual-fab implementation, $a_{R}=0.05$, $a_{L}=0.15$, $a_{G}=0.30$, ρ=0.075, $\lambda_{c}=0.15$, τ=0.58, S=3, and κ=3. The intervention cost is $c\left(u\right)=\sum_{k=1}^{6}r_{k}u_{k}^{2}$ with r=(1.25,1.00,0.82,1.35,1.15,0.90) and c(u)≤3.50. These constants define the baseline PG-RCA fit and are exported by the executable package. They are dimensionless benchmark settings for the synthetic study rather than calibrated proprietary recipe constants. They were fixed before evaluation on the held-out test sets, and no held-out labels were used to tune them. Their role is to create a nondegenerate benchmark with measurable cross-stage coupling, intervention cost, and tail risk; industrial use would require process-specific identification and physical-unit calibration. A one-at-a-time robustness audit over ±20% for $a_{R}$, $a_{L}$, $a_{G}$, $\lambda_{c}$, τ, and κ, together with a ±10% global disturbance-scale audit, is reported in Section 3.4; perturbations of the direct-coupling matrix *B* are reported in the graph-identification audit in the same section. Equation (36) is a smooth, scenario-restricted implementation score motivated by the certificate; it is not itself an upper probability bound. The exact certificate in Equation (33) must be reported separately when the qualification domain, ambiguity radius, and margin sensitivity are sufficiently informative.

**Proposition** **2** (Scenario-envelope approximation)**.**
*For any real scenario scores $r_{1},\ldots ,r_{S}$ and κ>0,*

(38) $$\max_{1\leq s\leq S}r_{s}\leq \frac{1}{\kappa }\log\sum_{s=1}^{S}e^{\kappa r_{s}}\leq \max_{1\leq s\leq S}r_{s}+\frac{\log S}{\kappa }.$$

*Consequently, minimizing Equation (35) minimizes an explicit additive approximation to the worst prescribed scenario.*

**Proof.**  Let $r_{\max}=\max_{s}r_{s}$. Because the sum contains a term equal to $e^{\kappa r_{\max}}$,

(39) $$\sum_{s}e^{\kappa r_{s}}\geq e^{\kappa r_{\max}},$$

which gives the lower inequality after applying $\kappa^{-1}\log\left(\;\cdot \;\right)$. Moreover, every term is at most $e^{\kappa r_{\max}}$, and hence,

(40) $$\sum_{s}e^{\kappa r_{s}}\leq Se^{\kappa r_{\max}}.$$

Taking the logarithm and dividing by κ gives the upper inequality. The final statement follows because the uniform upper bound log(S)/κ is independent of *u* once *S* and κ are fixed.    □

For $\bar{\alpha }\in \left(0,1\right)$, let $v\left(u\right)={\left[\sum_{k}\nu_{k}^{2}\left(u\right)\right]}^{1/2}$, and define the sub-Gaussian safety constraint

(41) $$g_{0}\left(u\right)=\sqrt{2\log(1/\bar{\alpha })}\;v\left(u\right)-\mu_{M}\left(u\right).$$

Whenever v(u)>0, the condition $g_{0}\left(u\right)\leq 0$ implies $C_{\mathrm{SG}}\left(u\right)\leq \bar{\alpha }$. A sufficient condition for convexity of $g_{0}$ is that *v* is convex and $\mu_{M}$ is concave on the qualified domain; otherwise, convexity must be verified or enforced by a local convex surrogate before applying the convergence theorem. For the nonlinear virtual-fab equations used later, these global convexity conditions are not asserted. The reported numerical policy is obtained instead from the separately defined operational score in Equation (36) by multi-start SQP; consequently, Theorem 4 is a conditional result for the exact convex certificate model and is not used as a proof of global convergence of the virtual-fab SQP run. Let $g_{q}=h_{q}$ for q=1,…,Q, where these functions represent process-window, thermal-budget, throughput, and tool-capability constraints. The reliability-oriented control problem is

(42) $$\begin{array}{cc} \min_{u\in U}\; & J\left(u\right)=R\left(u\right)+\lambda_{c}\sum_{k=1}^{K}c_{k}\left(u_{k}\right), \end{array}$$

(43) $$\begin{array}{cc} \mathrm{subject}\;\mathrm{to}\;\; & g_{q}\left(u\right)\leq 0,\;q=0,1,\ldots ,Q. \end{array}$$

To make the allocation auditable, define normalized stage contributions

(44) $$\omega_{k}\left(u\right)=\frac{\nu_{k}^{2}\left(u\right)}{\sum_{j=1}^{K}\nu_{j}^{2}\left(u\right)},\;\alpha_{k}=\bar{\alpha }\frac{\omega_{k}^{\gamma }}{\sum_{j=1}^{K}\omega_{j}^{\gamma }},$$

where γ∈[0,1] controls the balance between equal allocation and sensitivity-weighted allocation. The budgets $\alpha_{k}$ do not assume independent failure events. They are diagnostic shares used to prioritize metrology and control effort.

#### 2.3.2. Projected Risk-Certificate Algorithm

The objective contains an inner scalar minimization over δ. For each iterate $u^{t}$, we solve that minimization on a compact interval and compute an envelope gradient. A projected update is then performed:

(45) $$u^{t+1}={\mathrm{Proj}}_{U}\;\left[u^{t}-\gamma_{t}\left(\nabla R\left(u^{t}\right)+\lambda_{c}\nabla c\left(u^{t}\right)+\sum_{q=0}^{Q}\lambda_{q}^{t}\nabla g_{q}\left(u^{t}\right)\right)\right],$$

with dual update

(46) $$\lambda_{q}^{t+1}={\mathrm{Proj}}_{\left[0,\Lambda_{q}\right]}\;\left[\lambda_{q}^{t}+\tau_{t}g_{q}\left(u^{t}\right)\right],\;q=0,1,\ldots ,Q.$$

Algorithm 1 summarizes the complete procedure, termed process-graph reliability certificate allocation (PG-RCA).

**Assumption** **4** (Optimization regularity)**.**
*On the feasible qualified domain, J is differentiable, $L_{J}$-smooth, and $\mu_{J}$-strongly convex. Each constraint $g_{q}$, including the safety constraint in Equation (41), is convex, continuously differentiable, and has an $L_{q}$-Lipschitz gradient. A Slater point exists, the dual projection bounds $\Lambda_{q}$ contain at least one optimal multiplier, and the inner minimizer $\delta^{★}\left(u\right)$ is unique and lies in the interior of its interval. For an optimal multiplier $\lambda^{★}$, define*

(47) $$L_{L}=L_{J}+\sum_{q=0}^{Q}\lambda_{q}^{★}L_{q}.$$

**Algorithm 1** Process-graph reliability certificate allocation
**Require:** Process graph G, wafer records ${\left\{\zeta_{i}\right\}}_{i=1}^{n}$, physical maps, qualified set U, risk limit $\bar{\alpha }$**Ensure:** Intervention vector $u^{★}$, stage budgets $\left\{\alpha_{k}\right\}$, reliability certificate  1:Initialize $u^{0}\in U$, dual variables $\lambda_{q}^{0}\geq 0$ for q=0,…,Q, and graph Jacobians  2:**for** t=0,1,…,T−1 **do**  3:      Propagate states and path operators using Equations (1) and (11)  4:      Evaluate margins $M(\zeta_{i},u^{t})$ and transported scales $\nu_{k}\left(u^{t}\right)$  5:      Minimize Equation (33) over $\delta \in [\delta_{\min},\delta_{\max}]$  6:      Compute the envelope gradient and update $u^{t+1}$ by Equation (45)  7:      Update all dual variables by Equation (46)  8:      **if** the objective and feasibility residuals satisfy stopping tolerances **then**  9:            **break**10:      **end if**11:
**end for**
12:For diminishing coupled steps, form ${\bar{u}}^{T}=A_{T}^{-1}\sum_{t=0}^{T-1}a_{t}u^{t}$; otherwise set ${\bar{u}}^{T}=u^{t+1}$13:Compute $\omega_{k}$, $\alpha_{k}$, $C_{\mathrm{SG}}$, and the Wasserstein certificate14:**return** $u^{★}\approx {\bar{u}}^{T}$ and the reliability report

**Theorem** **4** (Convergence of PG-RCA)**.**
*Under Assumption 4, fix an optimal multiplier $\lambda^{★}$ and apply the projected primal update with the exact envelope gradient and $0<\gamma \leq 2/(L_{L}+\mu_{J})$. Then*

(48) $${∥u^{t}-u^{★}∥}^{2}\leq {\left(1-\frac{2\gamma L_{L}\mu_{J}}{L_{L}+\mu_{J}}\right)}^{t}{∥u^{0}-u^{★}∥}^{2}.$$

*For the coupled projected recursion, use a common diminishing step $\gamma_{t}=\tau_{t}=a_{t}$ with $\sum_{t}a_{t}=\infty$ and $\sum_{t}a_{t}^{2}<\infty$. Let $A_{T}=\sum_{t=0}^{T-1}a_{t}$, and define the weighted averages ${\bar{u}}^{T}=A_{T}^{-1}\sum_{t=0}^{T-1}a_{t}u^{t}$ and ${\bar{\lambda }}^{T}=A_{T}^{-1}\sum_{t=0}^{T-1}a_{t}\lambda^{t}$. Then, the restricted saddle gap over $U\times \prod_{q=0}^{Q}\left[0,\Lambda_{q}\right]$ converges to zero. Consequently, ${\bar{u}}^{T}\to u^{★}$, every cluster point of ${\bar{\lambda }}^{T}$ is dual optimal, and ${\bar{\lambda }}^{T}$ converges when the dual optimum is unique.*

**Proof.**  Let $L\left(u,\lambda^{★}\right)=J\left(u\right)+\sum_{q=0}^{Q}\lambda_{q}^{★}g_{q}\left(u\right)$. Convexity of every $g_{q}$ preserves the $\mu_{J}$-strong convexity contributed by *J*, while Equation (47) gives an $L_{L}$-Lipschitz gradient. The standard interpolation inequality for a differentiable, strongly convex, smooth function therefore yields

(49) $$\begin{array}{cc} \; & \langle \nabla_{u}L\left(u,\lambda^{★}\right)-\nabla_{u}L\left(u^{★},\lambda^{★}\right),u-u^{★}\rangle \\ \; & \;\geq \frac{L_{L}\mu_{J}}{L_{L}+\mu_{J}}{∥u-u^{★}∥}^{2}+\frac{1}{L_{L}+\mu_{J}}{∥\nabla_{u}L\left(u,\lambda^{★}\right)-\nabla_{u}L\left(u^{★},\lambda^{★}\right)∥}^{2}. \end{array}$$

Because projection onto a closed convex set is non-expansive and $u^{★}$ satisfies the projected optimality condition,

(50) $$\begin{array}{cc} {∥u^{t+1}-u^{★}∥}^{2}\; & \leq {∥u^{t}-u^{★}∥}^{2}-2\gamma \langle \Delta_{t},u^{t}-u^{★}\rangle +\gamma^{2}{∥\Delta_{t}∥}^{2}, \end{array}$$

where $\Delta_{t}=\nabla_{u}L\left(u^{t},\lambda^{★}\right)-\nabla_{u}L\left(u^{★},\lambda^{★}\right)$. Substitution of Equation (49) into Equation (50) makes the coefficient of ${∥\Delta_{t}∥}^{2}$ nonpositive for the stated step-size range. Hence,

(51) $${∥u^{t+1}-u^{★}∥}^{2}\leq \left(1-\frac{2\gamma L_{L}\mu_{J}}{L_{L}+\mu_{J}}\right){∥u^{t}-u^{★}∥}^{2},$$

and iteration proves Equation (48).

For the coupled recursion, set $z^{t}=\left(u^{t},\lambda^{t}\right)$, $Z=U\times \prod_{q=0}^{Q}\left[0,\Lambda_{q}\right]$, and $F\left(z\right)=(\nabla_{u}L\left(u,\lambda \right),-g\left(u\right))$. Compactness and smoothness give $∥F(z)∥\leq G$ on Z. For any z=(v,λ)∈Z, non-expansiveness of projection gives

(52) $${∥z^{t+1}-z∥}^{2}\leq {∥z^{t}-z∥}^{2}-2a_{t}\langle F\left(z^{t}\right),z^{t}-z\rangle +a_{t}^{2}G^{2}.$$

Convexity in *u* and linearity in λ imply

(53) $$\langle F\left(z^{t}\right),z^{t}-z\rangle \geq L\left(u^{t},\lambda \right)-L\left(v,\lambda^{t}\right).$$

Summing Equations (52) and (53), dividing by $2A_{T}$, and using convexity yields, uniformly for *v* and λ,

(54) $$L\left({\bar{u}}^{T},\lambda \right)-L\left(v,{\bar{\lambda }}^{T}\right)\leq \frac{\mathrm{diam}{\left(Z\right)}^{2}+G^{2}\sum_{t=0}^{T-1}a_{t}^{2}}{2A_{T}}.$$

Taking the maximum over λ and the minimum over *v* proves that the restricted saddle gap vanishes. Because $L(\;\cdot \;,\lambda^{★})$ is $\mu_{J}$-strongly convex and $\left(u^{★},\lambda^{★}\right)$ is a saddle point, this gap is at least $\mu_{J}{∥{\bar{u}}^{T}-u^{★}∥}^{2}/2$; hence, ${\bar{u}}^{T}\to u^{★}$. Compactness gives cluster points of ${\bar{\lambda }}^{T}$. Passing to the limit in the zero-gap relation shows that each cluster point attains the dual optimum. If that optimum is unique, compactness and uniqueness force the entire averaged dual sequence to converge. □

#### 2.3.3. Computational Scaling

Let $d=\sum_{k}d_{k}$, m=|E|, and *n* be the number of wafers. Sparse forward propagation requires O(n(d+m)) operations when local Jacobians are stored in sparse form. Reverse accumulation of margin sensitivities has the same order. In the reported implementation, the scalar optimization over δ is computationally small relative to state propagation. The scenario-restricted implementation evaluates *S* forward passes and therefore has cost O(Sn(d+m)); in the reported study, S=3. If full covariance matrices are required, the cost can grow to $O(nd^{2})$; however, low-rank or diagonal-plus-low-rank representations reduce this cost. The algorithm therefore scales with the physical sparsity of the fabrication graph rather than with the number of all possible stage interactions.

**Remark** **3.** *PG-RCA is designed as an engineering decision layer, not as a replacement for run-to-run control. A fast controller may continue to regulate individual tools, while PG-RCA operates at a slower cadence to update risk budgets, process windows, and metrology priorities. This separation preserves tool-level responsiveness and adds cross-stage reliability awareness. It also permits conservative deployment because only bounded intervention intensities are recommended within already qualified operating regions.*

### 2.4. Virtual-Fab Simulation Design

#### 2.4.1. Reproducible Physics-Guided Data Generator

We constructed a reproducible virtual fab to evaluate the theory without using confidential manufacturing data. The generator is intentionally synthetic and is designed to expose causal propagation, distribution shift, and tail behavior under controlled conditions. Six normalized stage innovations enter the process graph and share a latent common-mode component. A strictly lower-triangular direct-coupling matrix *B* specifies one-step edges, while ${\left(I-B\right)}^{-1}$ transports each innovation through the sum of all directed paths. The final defect energy includes stagewise quadratic terms and selected interactions for deposition–lithography, lithography–etch, etch–CMP, implantation–annealing, and annealing–CMP. Electrical drift and a downstream lifetime diagnostic are then computed from distinct but coupled functions, preventing the failure label from becoming a trivial transform of any single process variable.

The stage settings used by the generator are summarized in Table 3. The table reports disturbance scale, principal physical output, representative direct coupling, and allowable normalized adjustment. These dimensionless values were selected to create plausible relative sensitivities rather than to reproduce a proprietary technology node. The accompanying reproducibility package also records the exact matrix, random seeds, and executable equations.

Table 3 shows that lithography, etching, and CMP combine comparatively large disturbance scales with high downstream influence. Deposition has a smaller direct reliability coefficient, yet its location at the graph entrance allows its fluctuations to reach several later stages. Implantation has the lowest noise scale but remains important because annealing can convert a small dose or damage-profile error into activation and diffusion error. The control interval is identical across stages only because adjustments are normalized; physical implementations would map the normalized variable to stage-specific temperature, dose, flow, pressure, or timing ranges.

To ensure manuscript–code consistency, the exact normalized generator is specified below. With stage order (deposition, lithography, etching, implantation, annealing, and CMP), the direct-coupling matrix is

(55) $$B=\left[\begin{array}{cccccc} 0 & 0 & 0 & 0 & 0 & 0\\ 0.24 & 0 & 0 & 0 & 0 & 0\\ 0.10 & 0.35 & 0 & 0 & 0 & 0\\ 0 & 0.05 & 0.21 & 0 & 0 & 0\\ 0.065 & 0 & 0.10 & 0.29 & 0 & 0\\ 0.12 & 0 & 0.27 & 0 & 0.26 & 0 \end{array}\right],\;T={\left(I-B\right)}^{-1}.$$

Define the stage-scale vector and componentwise clipping bound as

σ=(0.62,0.70,0.66,0.55,0.58,0.73),b=3.35σ+0.951.

For each nominal wafer *i*, draw the following variables independently across wafers:

(56) $$c_{i}∼N\left(0,0.35^{2}\right),\;\varepsilon_{i}∼N\left(0,\mathrm{diag}\left(\sigma^{2}\right)\right),\;\xi_{i}={\mathrm{clip}}_{\left[-b,b\right]}\;\left(\varepsilon_{i}+c_{i}1\right),$$

where clipping is componentwise. All numerical experiments were implemented in Python 3.14.7 using NumPy 2.3.5 for array operations and random-number generation; the executable environment and dependencies are included in the accompanying reproducibility package. NumPy’s PCG64 generator is initialized with seed 20260806 for the 2400-wafer training set, 20260807 for the independent 6000-wafer nominal test set, and 20260808 for the independent 6000-wafer stress-base set. The executable instantiates PCG64 explicitly rather than relying on NumPy’s potentially version-dependent default bit generator. For stress intensity a≥0, the exact nested shift is

(57) $$\xi_{i}^{\left(a\right)}={\mathrm{clip}}_{\left[-b,b\right]}\;\left[\left(1+0.35a\right)\xi_{i}+0.15a\;\mathrm{sign}\left(\xi_{i}\right)\right].$$

The mild, reference, and severe held-out stress sets use a=0.35, 0.65, and 0.90, respectively; the two deterministic ambiguity representatives used during optimization apply Equation (57) to the training innovations at a=0.35 and 0.65. Hence, no held-out labels enter policy fitting. The executable code, synthetic data, processed audit tables are provided in the Supplementary Materials.

For a column innovation vector ξ and normalized control *u*, the transported state is

(58) x=Tdiag(1−0.55u)ξ.

The synthetic defect and electrical terms used by the executable package are

(59) $$\begin{array}{cc} D_{\mathrm{syn}}\left(x\right) & =\sum_{k=1}^{6}w_{k}x_{k}^{2}+\sum_{(i,j)\in I}\eta_{ij}\left|x_{i}x_{j}\right|, \end{array}$$

(60) $$\begin{array}{cc} P_{\mathrm{syn}}\left(x\right) & =\sum_{k=1}^{6}q_{k}|x_{k}\left|+0.17\right|x_{2}x_{3}|, \end{array}$$

(61) $$\begin{array}{cc} M_{\mathrm{syn}}\left(x\right) & =8.35-D_{\mathrm{syn}}\left(x\right)-0.72P_{\mathrm{syn}}\left(x\right), \end{array}$$

where w=(0.72,0.95,1.13,0.64,0.79,1.06), q=(0.35,0.58,0.76,0.29,0.42,0.60), and I={(1,2),(2,3),(3,6),(4,5),(5,6)} has coefficients (0.18,0.34,0.29,0.20,0.17) in the same order. The absolute-value terms are locally Lipschitz and are nondifferentiable only on zero-valued factor surfaces. Under the continuous innovation model, these surfaces have probability zero. The theoretical pathwise gradients are therefore evaluated almost surely away from the kinks; when a smooth derivative is required in another implementation, |a| may be replaced locally by $\sqrt{a^{2}+\epsilon^{2}}$ with a documented small ϵ>0. The diagnostic defect-density output is $0.021+0.0105D_{\mathrm{syn}}+0.004{\left(x_{3}+x_{6}\right)}_{+}^{2}$. Thus, the virtual-fab failure label is exactly $M_{\mathrm{syn}}\leq 0$; the equivalent-cycle variable in Equation (63) is a downstream diagnostic and is not part of this label.

#### 2.4.2. Data Splits, Distribution Shift, and Baselines

The training set contains 2400 wafers, while nominal and shifted test sets contain 6000 wafers each. Correlated stage innovations are bounded by a documented qualification envelope and are attenuated by the intervention action before multiplication by the total path-sum transport matrix ${\left(I-B\right)}^{-1}$. This ordering is essential: an upstream adjustment then changes every descendant state rather than only its local output. The held-out shift increases each innovation magnitude through a nested transformation, so mild, reference, and severe stress sets are ordered wafer by wafer. Failures use M≤0. A smooth margin transform is retained only as an operational warning score; it is not interpreted as a calibrated failure probability.

Five optimized policies are evaluated: four comparators—independent-stage Monte Carlo, a physics-only allocation, a stage-local data-only linear surrogate, and a process-graph empirical-risk baseline—together with PG-RCA-SQP. The uncontrolled case is retained only as a diagnostic reference. Every method is re-optimized under identical box constraints and the same stage-weighted quadratic upper budget; no comparator uses a hand-selected control vector. The data-only surrogate is fitted to the observed negative-margin labels using only the six stage-local absolute innovation magnitudes after intervention attenuation; it does not use *B*, $T={\left(I-B\right)}^{-1}$, graph-derived features, quadratic terms, or cross-stage products. The process-graph empirical-risk baseline, by contrast, uses the same known transport graph as the virtual-fab generator but optimizes only the empirical tail with a weak path prior, without ambiguity representatives or the transported-sensitivity term. PG-RCA-SQP forms two deterministic qualification representatives from the training records, evaluates Equation (35), and adds the local-sensitivity and graph-path terms in Equation (36). For exact reproducibility, the cost-penalized comparator scores are $0.05r_{\mathrm{ind}}+0.15c\left(u\right)$ for independent-stage MC, $2p_{G}+0.15c\left(u\right)$ for physics-only, $0.05r_{\mathrm{lin}}+0.15c\left(u\right)$ for the stage-local data surrogate, and $0.05r_{\mathrm{emp}}+0.10p_{G}+0.15c\left(u\right)$ for the process-graph empirical-risk baseline; PG-RCA uses the constants stated below Equation (36). The matched-budget variants retain the corresponding non-cost score and impose c(u)=3.50. The held-out nominal and shifted sets are never used during optimization.

## 3. Results and Discussion

### 3.1. Pathwise Sensitivity and Lifetime Response

The first diagnostic examines whether the numerical generator reproduces the directed transport structure assumed by the theory. For source stage *j* and affected stage *k*, the plotted first-order score is proportional to

(62) $$H_{kj}=\left|T_{kj}\right|\left(1-0.55u_{j}\right)\;{\bar{g}}_{k}\;\sigma_{j},$$

where $T={\left(I-B\right)}^{-1}$, ${\bar{g}}_{k}$ is the sample mean of a componentwise triangle-inequality upper proxy for $|\partial M/\partial x_{k}|$, and $\sigma_{j}$ is the idiosyncratic stage-noise scale from Equation (56). The common-mode term induces cross-stage covariance and is therefore not assigned to a single source column in this diagnostic heatmap; it remains represented by the covariance terms in Equation (15). The matrix is normalized by its largest entry only for visualization. Thus, an off-diagonal cell represents inherited downstream sensitivity and not a second application of the transport matrix.

**Figure 4:**
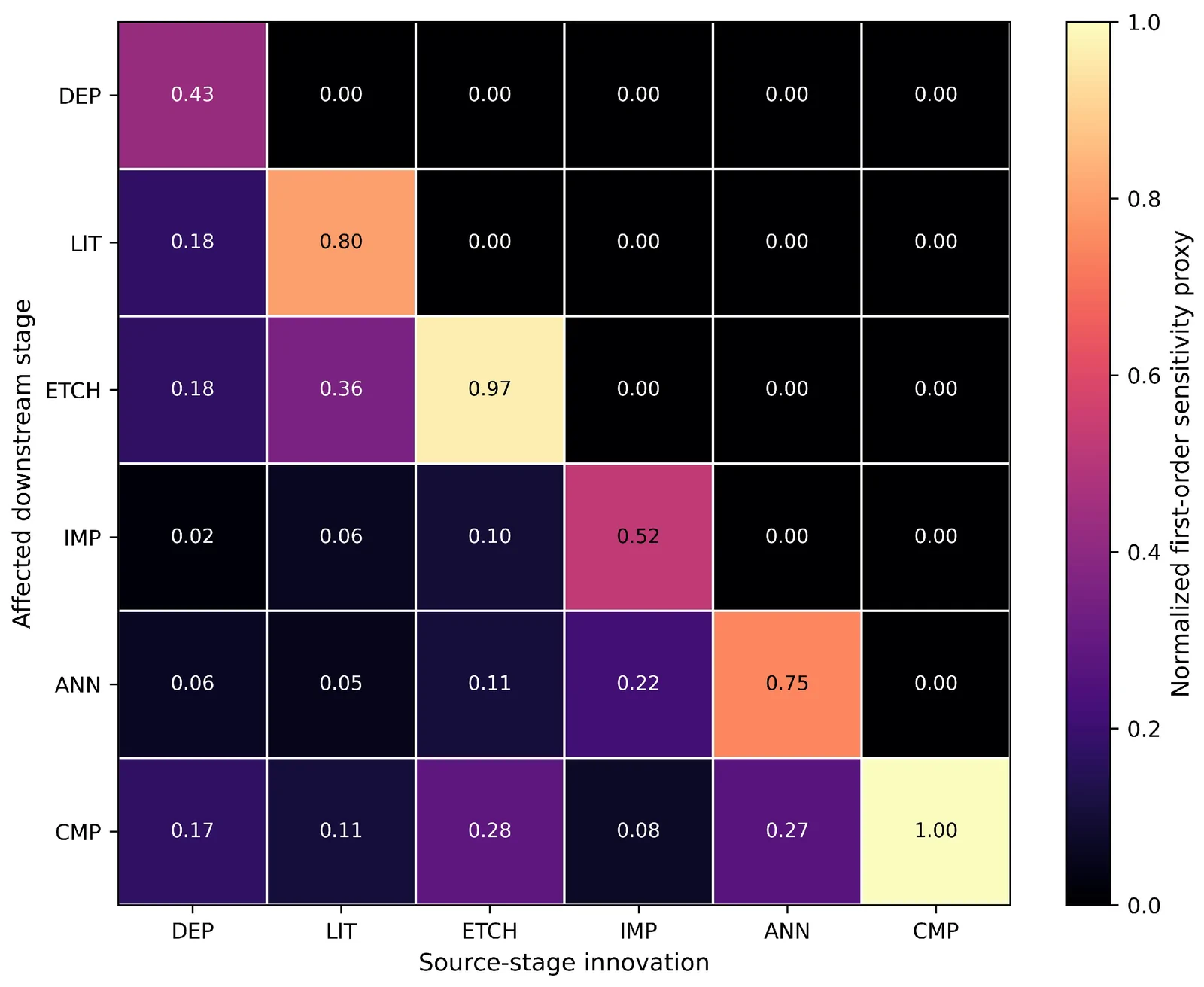
Normalized first-order cross-stage sensitivity.

Figure 4 shows strong diagonal response together with physically interpretable inherited effects. Lithography contributes 0.36 of the normalized sensitivity at etching, while the final CMP state receives substantial inherited contributions from etching (0.28) and annealing (0.27), in addition to its unit-normalized local term. Deposition also remains visible at CMP (0.17) because it lies upstream of several paths. These values are not probabilities or a full variance decomposition; they are normalized conservative first-order sensitivity proxies used to identify where metrology or tighter control can protect downstream reliability.

The virtual-fab lifetime variable is deliberately an equivalent-cycle index rather than a calibrated device lifetime. For the nominal synthetic model,

(63) $$T_{\mathrm{eq}}=\exp\;\left[12.3+0.62M-0.16{\left(x_{5}\right)}_{+}^{2}\right],\;T_{\mathrm{eq}}\left(s\right)=T_{\mathrm{eq}}\left(1\right)s^{-4.2},$$

where $x_{5}$ is the normalized annealing state and *s* is an imposed accelerated-stress multiplier. Equation (63) is the exact expression evaluated by the executable code; its constants are synthetic and are not fitted to a proprietary technology node or a physical acceleration law.

**Figure 5:**
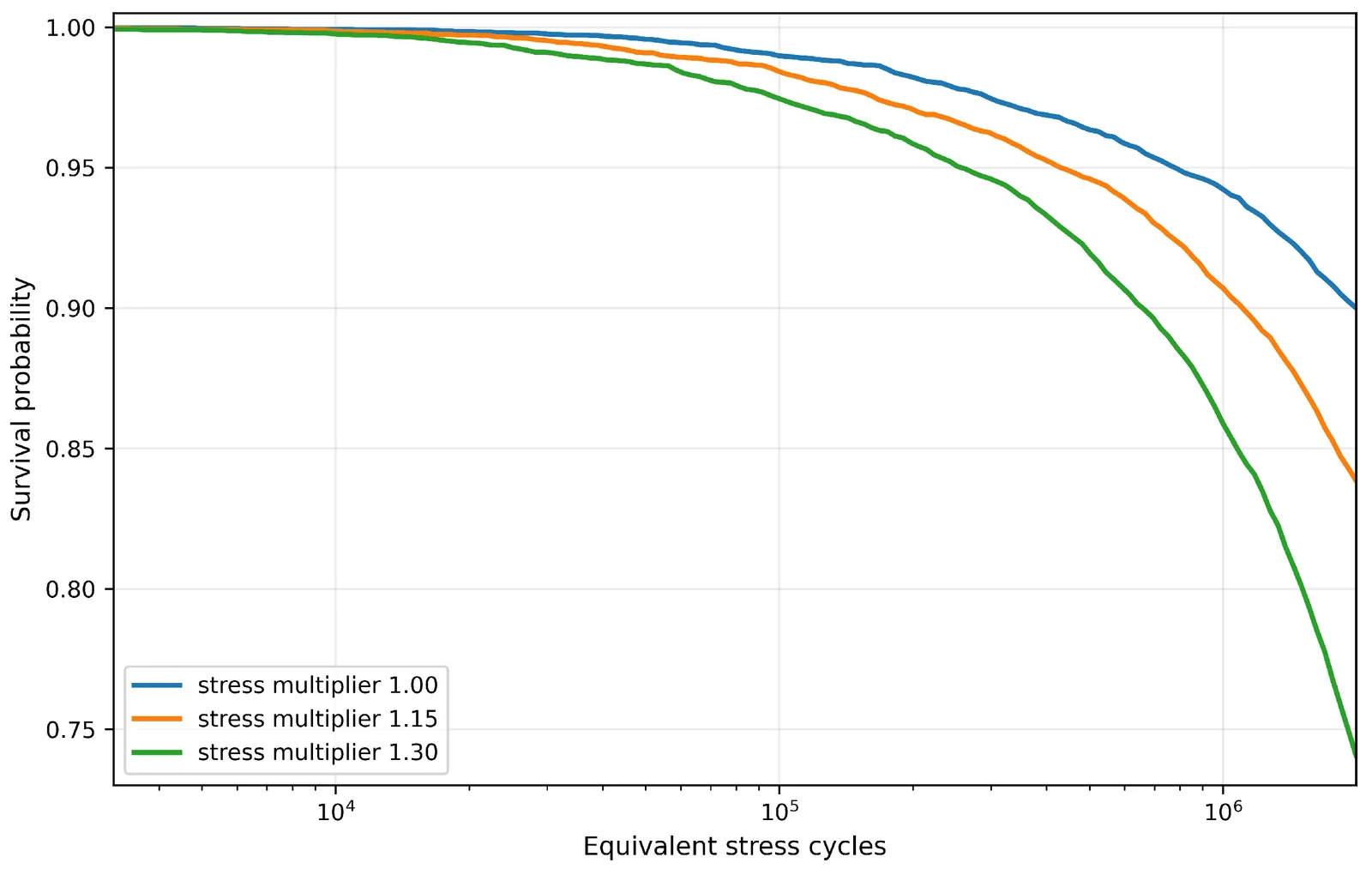
Synthetic accelerated-stress survival response.

Figure 5 confirms the intended monotone stress ordering. At approximately $2.0\times 10^{6}$ equivalent cycles, the empirical survival fractions are 0.900, 0.839, and 0.741 for multipliers 1.00, 1.15, and 1.30, respectively. The curves therefore confirm the intended internal coupling among the reliability margin, annealing damage, and the lifetime index, but they should not be interpreted as measured wafer-level survival. Their purpose is mechanism testing under known ground truth and reproducible stress ordering.

### 3.2. Comparative Reliability Performance and Matched-Budget Audit

Normalized intervention intensities are constrained to [0,0.85] with an intervention budget of 3.50. In the virtual fab, $u_{k}=0$ means no mitigation and larger $u_{k}$ attenuates the corresponding stage innovation before graph transport; $u_{k}$ is therefore a dimensionless control-effort variable rather than a signed physical set-point offset. The PG-RCA-SQP solution is ${\left(0.805,0.850,0.850,0.509,0.572,0.850\right)}^{⊤}$ from deposition through CMP. The six-dimensional operational problem uses the constants reported in Section 2.3, including S=3, κ=3, and τ=0.58. It is solved from eight deterministic or seeded starts by constrained sequential quadratic programming; every start returned a feasible solution, and the best objective was retained.

Because an upper budget does not force different cost-penalized objectives to use the same amount of control, the primary comparison can confound modeling differences with voluntary control effort. We therefore report two comparisons in Table 4. The primary design retains the common cost penalty and common upper budget. The matched-budget audit then re-optimizes every method with the intervention cost fixed at 3.50. This second comparison prevents differences in voluntary control effort from being mistaken for intrinsic algorithmic superiority.

Under the cost-penalized formulation, PG-RCA-SQP has the lowest nominal and shifted empirical failure and the lowest DKW upper bound, but it also uses the full intervention budget. Once every method is constrained to the same cost of 3.50, shifted failures lie in the narrow 81.2–82.5 kppm range; the physics-only policy is numerically lowest by six failed wafers out of 6000 relative to PG-RCA-SQP. Because all methods are evaluated on the same shifted wafers, we also performed exact paired tests on discordant failure indicators. Relative to PG-RCA-SQP, the two-sided exact McNemar/binomial *p*-values are 0.860, 0.418, 1.000, and 1.000 for the independent-stage, physics-only, data-only, and process-graph empirical-risk policies, respectively. These tests provide no evidence of a method difference at the 0.05 level in this synthetic matched-budget experiment. Accordingly, the large 65.8% reduction relative to the original independent-stage policy is a joint consequence of risk modeling and more aggressive control use, not evidence of unconditional method dominance.

**Figure 6:**
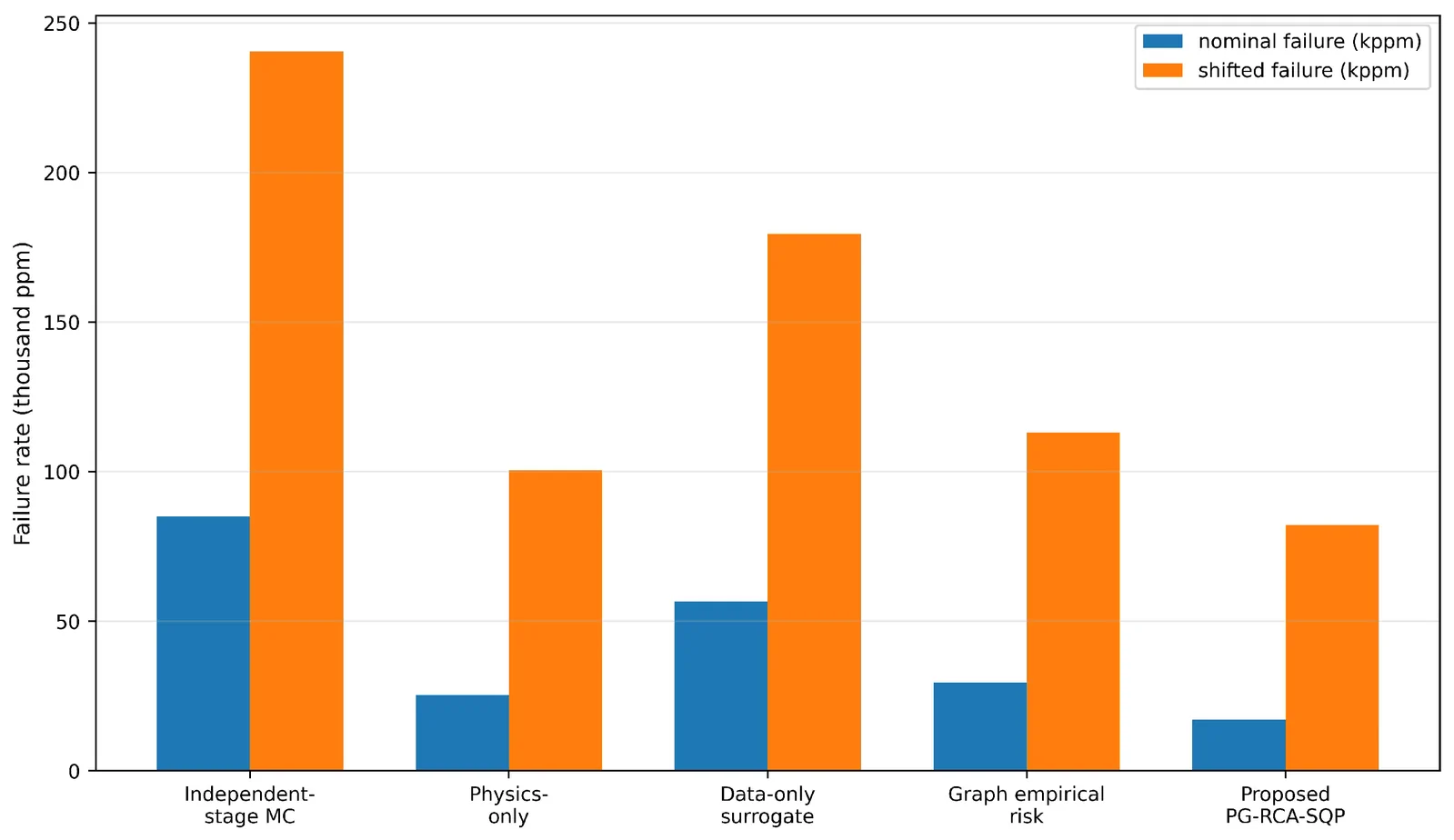
Cost-penalized failure comparison under the common upper budget.

Figure 6 visualizes the primary cost-penalized comparison. The proposed policy reduces nominal failure from 85.0 to 17.0 kppm and reference-shift failure from 240.5 to 82.2 kppm relative to the independent-stage policy. The matched-budget column in Table 4 is essential context for this figure: after control effort is equalized, the optimized policies differ by only a few failures among 6000 shifted wafers in this synthetic test. The practical value of PG-RCA is therefore its auditable pathwise risk attribution and certificate-compatible allocation, not a claim that it must always beat simpler policies at equal intervention intensity.

### 3.3. Stress Robustness and Numerical Certification

Table 5 separates data-scarcity and distribution-shift checks. For the first three rows, PG-RCA-SQP is refitted on nested training subsets and evaluated on the same independent nominal test set. Each subset fit uses only data-independent deterministic/PCG64 multi-start initializations; the full-2400-wafer solution is not used to initialize the 600- or 1200-wafer fits. For the final three rows, the full 2400-wafer training policy is held fixed while the independent stress set is transformed with increasing shift intensity. The latter construction is nested wafer by wafer, so monotonicity is a designed validation property rather than a post hoc selection.

The sample-size rows indicate a modest improvement from 20.0 to 17.0 kppm as the optimization set grows from 600 to 2400 wafers. The stress rows increase monotonically from 41.7 to 120.7 kppm, consistent with the intended nested-shift construction. These values are empirical failure rates, not Wasserstein certificates. For policy qualification, the independent 6000-wafer test set also permits the distribution-free DKW bound in Corollary 1; at 95% confidence, the proposed policy has a nominal upper bound of 34.5 kppm and a reference-shift upper bound of 99.7 kppm.

**Figure 7:**
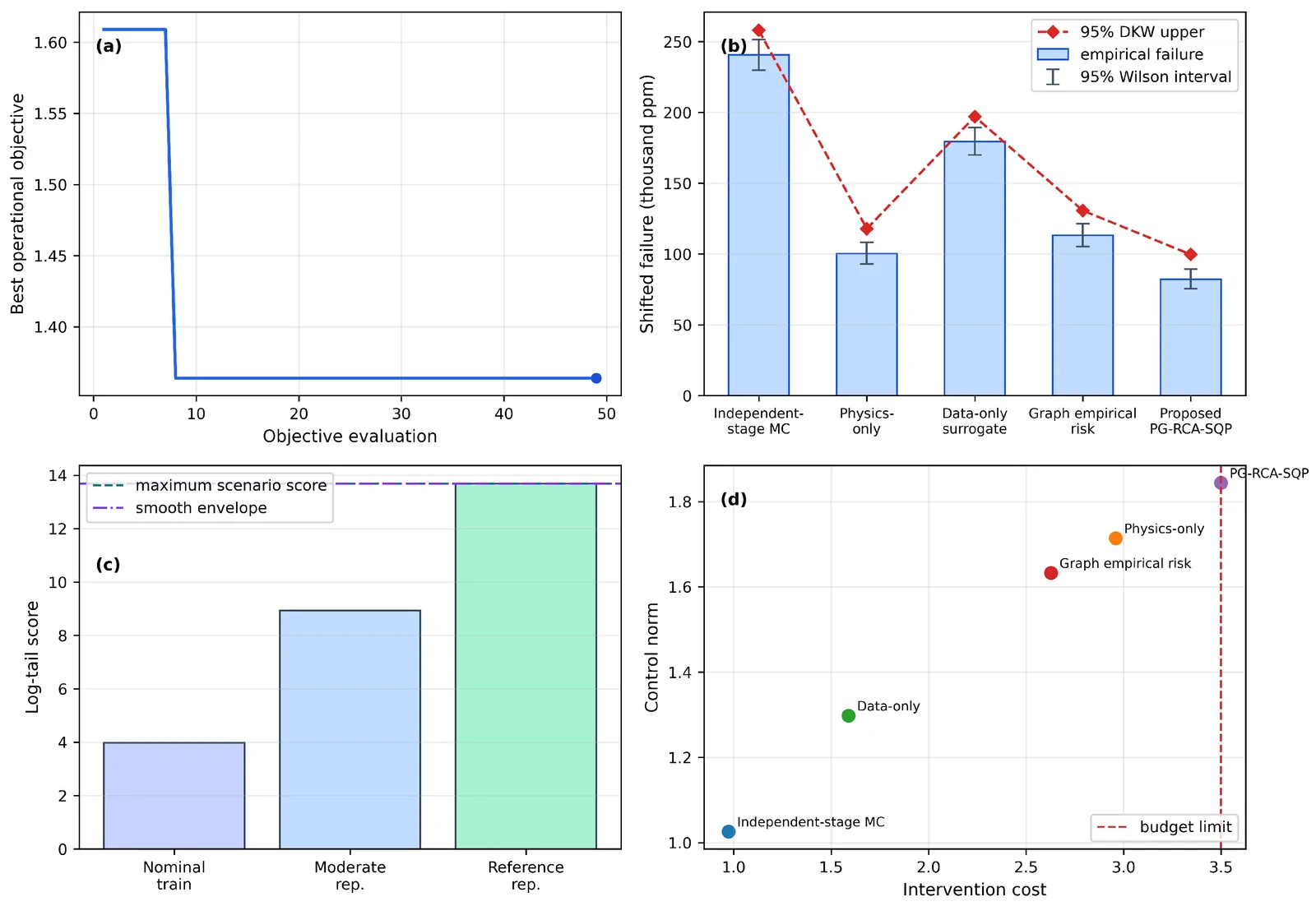
Optimization, certification, and scenario diagnostics: (**a**) convergence of the best operational objective over objective evaluations; (**b**) shifted empirical failure, 95% Wilson intervals, and 95% DKW upper bounds for the compared methods; (**c**) log-tail scores for the nominal-training, moderate-representative, and reference-representative scenarios, together with the maximum-scenario score and smooth-envelope guides; (**d**) intervention cost versus control norm for the compared methods, with the budget limit shown by the dashed line.

Figure 7 provides the optimization and certification dashboard. Panel (a) reports the running-best operational objective over SQP evaluations. Panel (b) compares shifted empirical failure with its 95% Wilson interval and the 95% DKW upper bound evaluated at M=0 for every optimized method. Panel (c) audits the smooth scenario envelope, and panel (d) explicitly displays the unequal intervention costs that motivated the matched-budget analysis above.

Across eight starts, the retained SQP run decreases rapidly during early objective evaluations and then stabilizes; all starts satisfy the box and quadratic-budget constraints. This trace confirms numerical termination of the implemented finite-dimensional problem but does not substitute for the projected-algorithm proof. The scenario envelope differs from the maximum representative-scenario score by only $2.1\times 10^{-7}$, well below the log-sum-exp bound log(3)/3≈0.366. Panel (d) also makes the fairness limitation visible: several baseline policies stop far below the 3.50 budget under the common cost penalty, which is why the separate matched-budget audit is required.

### 3.4. Finite-Sample, Topology, Hyperparameter, and Flow-Length Audits

#### 3.4.1. Finite-Sample Bound Comparison

The independent 6000-wafer test sets make it possible to compare the uniform DKW certificate with threshold-specific binomial limits. Table 6 reports the proposed fixed policy. For the shifted set, the empirical failure is 82.2 kppm; the 95% Wilson, exact Clopper–Pearson, and percentile-bootstrap upper endpoints are 89.4, 89.4, and 89.2 kppm, respectively, whereas the DKW upper limit is 99.7 kppm. The same ordering is visible on the nominal set. Thus, DKW is deliberately conservative for a single threshold, but its approximately 10.3 kppm extra headroom over the exact binomial upper limit buys uniform CDF coverage. Operational qualification can therefore report DKW when threshold flexibility is important and use Wilson or Clopper–Pearson when the failure threshold is fixed in advance.

#### 3.4.2. Graph-Identification and Hyperparameter Sensitivity

To test dependence on the assumed process graph, each topology variant was re-optimized at exactly the same intervention cost, 3.50, and then evaluated on held-out wafers generated by the true graph. Table 7 shows that deleting the strongest lithography–etch edge, deleting that edge together with annealing–CMP, adding a spurious lithography–CMP edge, or scaling every direct gain by ±20% changes the selected control vector but leaves shifted failure between 81.2 and 82.3 kppm in this particular virtual fab. This narrow range should not be interpreted as proof of topology robustness: the tested perturbations are local and the data generator is still structurally related to the assumed family. The experiment establishes only that moderate graph errors do not destabilize this synthetic equal-budget solution; unknown topology in an industrial fab remains an open identification problem.

The one-at-a-time parameter audit likewise separates tuning sensitivity from data-generation severity. Varying $a_{R}$, $a_{L}$, $a_{G}$, $\lambda_{c}$, τ, and κ by ±20% changes shifted failure within 82.0–85.7 kppm; the largest control-vector change is 0.054 in Euclidean norm. The direct-coupling matrix *B* is tested separately above through missing, extra, and ±20% edge-gain perturbations. Globally scaling the complete stage-innovation vector σ by −10% and +10% changes shifted failure to 46.0 and 120.5 kppm, respectively. This larger change is expected because it modifies physical disturbance severity rather than only an optimizer weight. Together, the *B*, σ, and objective-constant audits cover the principal constants highlighted by Reviewer 1. They confirm that the reported baseline was not selected from held-out test labels while also showing that reliability remains materially dependent on physical process-noise calibration.

#### 3.4.3. Manufacturing-Flow Length

Flow length affects both computation and uncertainty amplification. To isolate this effect without inventing uncalibrated defect models for additional unit operations, we extended the sparse acyclic transport graph synthetically while keeping local gains fixed at 0.25 for the preceding stage and 0.08 for the two-stage skip connection. As the chain grows from 3 to 24 stages, the number of edges rises from 3 to 45, while the maximum column $\ell_{1}$ transport gain increases from 1.393 to 1.493 and then nearly saturates. The spectral norm increases from 1.229 to 1.479. For sparse stable couplings of this magnitude, longer flows therefore increase accumulated path influence but do not produce explosive amplification. Computationally, the sparse forward/reverse passes remain O(n(d+m)), as stated above. Denser graphs, stronger gains, rework loops, or chamber-memory states can grow much faster and require the cyclic or state-augmented extensions discussed below.

### 3.5. Ablation and Mechanism Analysis

The ablation study requires the same effort control as the main comparator study. Each variant therefore has two reported results, as summarized in Table 8. The cost-penalized column preserves the stated objective and common upper budget, while the matched-budget column re-optimizes the same variant at an intervention cost of exactly 3.50. Removing cross-stage edges suppresses path propagation inside the optimization model while the held-out wafers are still generated and evaluated with the full graph. “No interaction physics” removes the nonlinear PSPR interactions, “No sensitivity penalty” removes only the transported local-sensitivity term, and “No tail allocation” replaces the exponential-tail objective with a worst-scenario mean-margin objective.

These two columns materially change the interpretation of the mechanism study. Under the original cost penalty, removing tail allocation or graph transport greatly increases failure, but these variants also choose much less intervention effort. After fixing every variant at cost 3.50, all shifted failure rates lie between 80.7 and 82.2 kppm, and none of the removed-mechanism variants is worse than the full method on this finite synthetic test. The equal-effort experiment therefore does not identify a measurable failure-rate benefit for any individual penalty or graph term. The graph and sensitivity components directly provide structured attribution and compatibility with the analytical certificate. However, establishing their incremental predictive or control benefit requires a richer process distribution, independently identified graph uncertainty, or industrial data.

**Figure 8:**
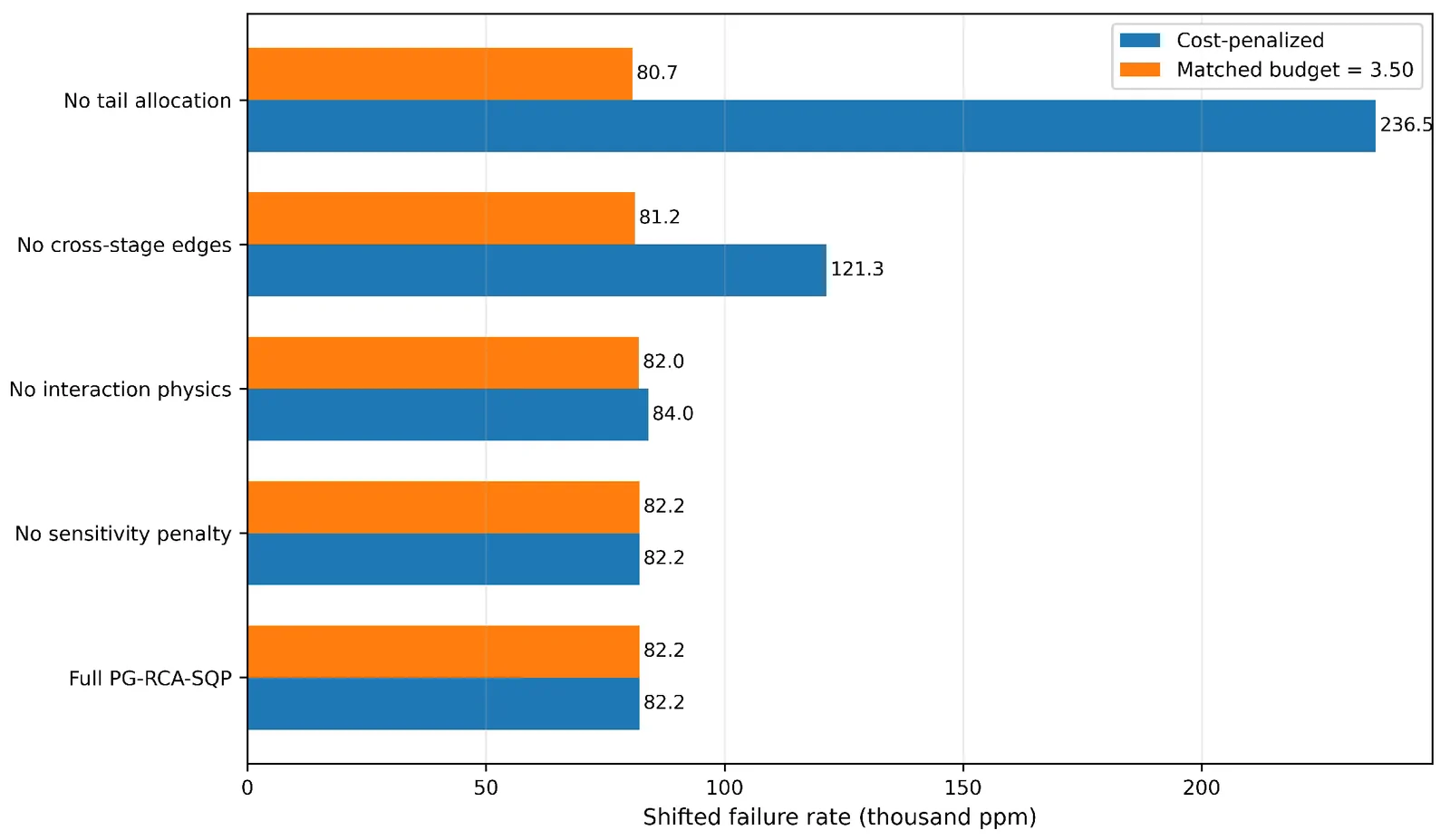
Ablation comparison before and after intervention-cost matching.

Figure 8 illustrates this confounding: the large gaps in the cost-penalized bars collapse once intervention effort is equalized. With 6000 shifted wafers, one failure corresponds to approximately 0.167 kppm; the full and matched ablations differ by at most nine failures. These small numerical differences should not be interpreted as evidence that removing a mechanism improves reliability. Instead, the matched-budget result sets a stricter evidentiary boundary: this synthetic experiment demonstrates computational consistency and attribution behavior, but it does not empirically isolate the causal value of each regularization component.

### 3.6. Engineering Interpretation and Limitations

The results motivate three practical uses. First, the diagonal term $s_{k}^{⊤}\Sigma_{kk}s_{k}$, interpreted together with the cross-stage terms $s_{k}^{⊤}\Sigma_{k\ell }s_{\ell }$, can help prioritize metrology investments. A stage with a high local or correlated transported contribution deserves denser sampling even if its local defect rate is small. Second, risk budgets can guide recipe qualification by showing whether a proposed change consumes margin locally or through a downstream interaction. Third, the margin can connect in-line measurements to lifetime screens, supporting earlier containment than would be possible from final electrical testing alone.

Several limitations remain. The present experiments use a low-dimensional normalized generator and a known acyclic graph. The new topology audit evaluates several missing, extra, and perturbed edges, but it does not establish performance when the graph must be learned from noisy fab records. In practice, graph-identification error can bias both the attributed upstream source and the transported sensitivity magnitude; missing edges tend to understate downstream exposure, whereas spurious edges can divert metrology or control effort. The comparator study also assumes access to the synthetic stage innovations used to build the stage-local data surrogate, whereas real fabs may observe only noisy, censored, or indirect proxies. Rework loops violate the single-pass DAG representation unless a rework visit is unrolled as a new state; chamber histories and maintenance states create memory and latent tool-condition variables; mixed products require product-conditioned maps or hierarchical parameters; and delayed labels increase uncertainty in the link between in-line metrology and final electrical failure. Each omitted feature can reduce accuracy by creating an unmodeled dependency rather than merely adding random noise. Incorporating these variables should improve calibration only when they are measured and identified reliably; indiscriminate state expansion can instead increase variance and ambiguity-set size. The first-order path operator may be insufficient near a strongly nonlinear regime transition. Wasserstein ambiguity protects against moderate distribution change but cannot make arbitrary extrapolation safe. Equation (27) can still be conservative when the ambiguity radius or transported Lipschitz estimate is loose, while Equation (31) applies only to a fixed policy evaluated on an independent certification sample and does not cover model-form shift. The scenario score was therefore kept explicitly separate from a certified probability. Finally, any recipe recommendation must be mapped to physically qualified units and validated through designed experiments before production deployment.

Greater process granularity changes the state definition and edge physics rather than the mathematical architecture. For lithography, immersion and EUV should use different state descriptors for dose, focus, mask/reticle effects, stochastic resist behavior, and thermal/overlay mechanisms; for deposition, CVD, PVD, and ALD require different kinetic, transport, conformality, and precursor-history terms. Mature nodes may permit narrower, empirically stable parameter distributions, whereas advanced nodes can require additional stochastic terms for atomic-scale variability, patterning interactions, and tighter overlay budgets. The framework can accommodate these distinctions by splitting a generic stage into technology-specific nodes or by conditioning $f_{k}$, $G_{k}$, and the qualification domain on process family and node maturity. However, parameters must be re-identified for each technology; the normalized six-stage values in this study must not be transferred directly between immersion, EUV, CVD, PVD, ALD, or different technology nodes.

For practical model evaluation, the mathematical objects map to specific engineering questions: $T_{kj}$ asks how an upstream perturbation reaches a downstream state; $H_{kj}$ ranks where additional metrology can reduce uncertainty; the sub-Gaussian scale $\nu_{k}$ identifies stages that consume tail-risk budget; the independent-sample interval quantifies whether a fixed policy meets a failure target; and the optimized $u_{k}$ is only a normalized mitigation intensity that must be translated into a qualified recipe change. The equations are therefore not intended to be evaluated as abstract scores in isolation. A process engineer would use them together with the held-out margin distribution, path attribution, intervention cost, and certificate before deciding whether to run a split lot or designed experiment.

### 3.7. Practical Qualification Protocol

Production deployment can follow a four-gate protocol. At the mechanism gate, process engineers approve the graph topology, state definitions, and signs of the dominant couplings. At the data gate, each metrology stream is checked for sampling policy, tool matching, censoring, and time alignment; an unavailable stream is represented by a wider ambiguity radius rather than silent imputation. At the certificate gate, the target failure budget, confidence level, and permitted distribution-shift radius are fixed before recipe optimization. At the execution gate, the recommended intervention intensity is restricted to the previously qualified window and is first evaluated on monitor wafers or a split lot. This sequence separates model construction, statistical calibration, and physical authorization.

The certificate should also be monitored after deployment. For lot *t*, the realized margin distribution, independent-sample failure bound, and path contributions are compared with their qualification baselines. A warning is issued when the empirical failure frequency exceeds the certified envelope, when the Wasserstein distance from the reference data crosses its approved radius, or when a path contribution changes beyond a process-specific tolerance. Such a warning should not trigger an immediate large recipe change. Instead, the system freezes the current recommendation, identifies the stages responsible for the increase, and requests targeted metrology or designed experiments. This closed-loop governance positions the proposed optimizer as an auditable decision-support layer while preventing statistical confidence from being mistaken for unrestricted process authority.

**Remark** **4.** *The numerical gains should be interpreted alongside both matched-budget audits. Equalizing intervention cost largely removes the performance gap among optimized policies and also collapses the apparent cost-penalized ablation gaps. The synthetic evidence therefore supports the internal consistency, pathwise attribution, and certificate-compatible decision structure of PG-RCA, but it does not establish that each regularization component independently reduces failure at equal effort. Industrial value will depend on graph identification, trustworthy metrology, physically calibrated margins, and conservative qualification.*

## 4. Conclusions and Future Work

This paper introduced a process-graph reliability framework for advanced semiconductor material processing. Deposition, lithography, etching, implantation, annealing, and CMP were modeled as coupled stochastic transformations inside a unified process–structure–property–reliability map. A directed path operator converted local Jacobians into cross-stage uncertainty contributions. On this basis, a sub-Gaussian failure bound and a finite-data Wasserstein certificate were derived. A projected risk-allocation algorithm was analyzed for the exact certificate-based model, and a scenario-restricted multi-start SQP implementation optimized bounded intervention intensities for the virtual-fab study. Theoretical results established trajectory well-posedness, first-order covariance decomposition, non-asymptotic failure certification, and convergence of the projected PG-RCA recursion under the stated convexity and regularity conditions.

The reproducible virtual-fab experiments illustrate both the practical implications and the limitations of the framework. Under the cost-penalized design, PG-RCA-SQP achieved 98.30% nominal yield and 82.2 kppm reference-shift failure with a 95% DKW upper bound of 99.7 kppm. However, a matched-budget audit placed all optimized methods between 81.2 and 82.5 kppm, and exact paired tests provided no evidence of a matched-budget difference from PG-RCA-SQP at the 0.05 level. A second matched-budget audit of the ablations likewise collapsed the large cost-penalized mechanism gaps to 80.7–82.2 kppm. Accordingly, the empirical conclusion is deliberately limited: the framework provides reproducible cross-stage attribution and a certificate-compatible optimization structure, while equal-effort reliability advantages and componentwise causal gains remain to be established on richer or industrial data. Additional revision-stage audits show that moderate missing/extra-edge perturbations keep the equal-budget shifted failure within 81.2–82.3 kppm, objective-weight perturbations remain within 82.0–85.7 kppm, and threshold-specific binomial upper limits are materially tighter than the uniform DKW bound. These checks strengthen the interpretation of the method as an attribution and qualification framework, not as evidence of universal optimizer superiority.

Future work will focus on three directions. First, graph learning will be extended to partially observed fabs with rework loops, chamber memory, and hidden maintenance states while preserving physically admissible edge constraints. Second, the certificate will be generalized to nonlinear distribution shifts using sequential conformal calibration and mechanism-aware ambiguity sets that distinguish product, tool, and environment changes. Third, evaluation will extend to multi-product industrial data and accelerated-life measurements, including designed experiments to map normalized intervention intensities to qualified physical set-point changes and to calibrate the synthetic lifetime index against device-specific failure physics.

## Figures and Tables

**Table 1:** Positioning relative to representative recent methods.

Method	Primary Role	Physical Cross-Stage Paths	Finite-Sample Failure Bound	Equal-Effort Audit
Hierarchical virtual metrology [11]	Multimodal metrology prediction	Hierarchical data fusion	No	No
Digital twin with Bayesian optimization [14]	Process-control optimization	Digital twin process model	No	No
Ontology-driven Bayesian network [23]	Fault diagnosis and correction	Causal/semantic dependencies	No	No
Tensor process control [17]	Monitoring and control under disturbances	High-dimensional process dependence	No	No
This work	Reliability attribution and qualification	Ordered Jacobian products over fabrication paths	Sub-Gaussian, Wasserstein, and independent-sample bounds	Equal-cost comparator and ablation audits

**Table 2:** Principal symbols and definitions.

Symbol	Definition
G=(V,E)	Directed acyclic graph of material-processing stages
*K*	Number of processing stages
$x_{k}$	Normalized material and process state after stage *k*
$u_{k}$	Normalized intervention intensity at stage *k*
$\theta_{k}$	Physical parameters of stage *k*
$\varepsilon_{k}$	Random disturbance entering stage *k*
$f_{k}$	Physics-informed stage-transition map
$J_{k\leftarrow j}$	Jacobian transporting perturbations from stage *j* to *k*
$\Pi_{p}$	Ordered Jacobian product along directed path *p*
*z*	Final structure–property descriptor
D(z)	Nonnegative defect functional
P(z)	Electrical-property drift functional
*M*	Signed reliability margin; failure occurs when M≤0
$\ell_{\delta }\left(M\right)$	Smooth bounded failure majorant with width δ
${\hat{P}}_{n}$	Empirical distribution of observed wafers
$W_{1}$	First-order Wasserstein distance
$\rho_{n}\left(\beta \right)$	Data-dependent ambiguity radius at confidence 1−β
R(u)	Certified robust failure-risk objective
$\alpha_{k}$	Allocated stage-risk budget
$u^{★}$	Reliability-oriented optimal intervention vector

**Table 3:** Virtual-fab stage settings.

Stage	Noise Scale	Main Normalized Output	Strong Direct Edge	Control Range
Deposition	0.62	Thickness, stress, interface roughness	Deposition → lithography: 0.24	[0,0.85]
Lithography	0.70	Critical dimension, focus, overlay	Lithography → etching: 0.35	[0,0.85]
Etching	0.66	Sidewall angle, depth, plasma damage	Etching → CMP: 0.27	[0,0.85]
Implantation	0.55	Dose and damage-profile error	Implantation → annealing: 0.29	[0,0.85]
Annealing	0.58	Activation, diffusion, residual defects	Annealing → CMP: 0.26	[0,0.85]
CMP	0.73	Dishing, erosion, non-uniformity	Final structure and margin	[0,0.85]

**Table 4:** Reliability performance and matched-budget fairness audit.

Method	Nominal Yield (%)	Shifted Failure (kppm)	Shifted DKW Upper (kppm)	Cost	Matched-Budget Shifted (kppm)
Independent-stage MC	91.50	240.5	258.0	0.974	82.5
Physics-only	97.48	100.3	117.9	2.960	**81.2**
Data-only surrogate	94.33	179.5	197.0	1.589	82.0
Process-graph empirical risk	97.05	113.2	130.7	2.628	82.3
Proposed PG-RCA-SQP	**98.30**	**82.2**	**99.7**	3.500	82.2

**Table 5:** Data-scarcity and distribution-shift robustness.

Scenario	Training Wafers	Shift Index	Failure (kppm)
Small sample	600	0.00	20.0
Moderate sample	1200	0.00	19.7
Full sample	2400	0.00	17.0
Mild shift	2400	0.35	41.7
Reference shift	2400	0.65	82.2
Severe shift	2400	0.90	120.7

**Table 6:** Finite-sample upper limits for the fixed PG-RCA-SQP policy (95% confidence).

Test Set	Empirical	Wilson	Clopper–Pearson	Bootstrap	DKW
Nominal (kppm)	17.0	20.6	20.6	20.3	34.5
Reference shift (kppm)	82.2	89.4	89.4	89.2	99.7

**Table 7:** Graph-identification sensitivity at matched intervention cost.

Assumed Graph	$∥B_{\mathrm{assumed}}{-B∥}_{F}$	$∥u-u_{\mathrm{true}}{∥}_{2}$	Shifted Failure (kppm)
True graph	0.000	0.000	82.2
Missing LIT→ETCH	0.350	0.114	81.2
Missing LIT→ETCH and ANN→CMP	0.436	0.055	82.3
Extra LIT→CMP edge	0.150	0.027	82.0
All edge gains −20%	0.140	0.047	81.5
All edge gains +20%	0.140	0.054	82.0

**Table 8:** Ablation results before and after equalizing intervention effort.

Variant	Cost-Penalized Cost	Cost-Penalized Shifted Failure (kppm)	Matched-Budget Shifted Failure (kppm)
Full PG-RCA-SQP	3.500	82.2	82.2
No cross-stage edges	2.470	121.3	81.2
No interaction physics	3.420	84.0	82.0
No sensitivity penalty	3.500	82.2	82.2
No tail allocation	0.980	236.5	**80.7**

## Data Availability

All synthetic data, executable code, processed result tables, and figure-generation resources required to reproduce the reported virtual-fab simulations are provided in the accompanying reproducibility package. No confidential production data were used.

## Supplementary Materials

The following supporting information can be downloaded at https://www.mdpi.com/article/10.3390/mi17101131/s1, The accompanying reproducibility package contains the executable Python code, four synthetic data files, processed result tables (including topology, hyperparameter, finite-sample, and flow-length audits).
